# Assessing age-dependent multi-task functional co-activation changes using measures of task-potency

**DOI:** 10.1016/j.dcn.2017.11.011

**Published:** 2017-12-05

**Authors:** Roselyne J. Chauvin, Maarten Mennes, Jan K. Buitelaar, Christian F. Beckmann

**Affiliations:** a Radboud University Medical Center, Department of Cognitive Neuroscience, Nijmegen, The Netherlands; b Donders Institute for Brain, Cognition and Behaviour, Radboud University Nijmegen, Nijmegen, The Netherlands; c Centre for Functional MRI of the Brain (FMRIB), University of Oxford, Oxford, United Kingdom

**Keywords:** Functional connectivity, Task fMRI, Resting state

## Abstract

It is being hypothesised that the developing adolescent brain is increasingly enlisting long-range connectivity, allowing improved communication between spatially distant brain regions. The developmental trajectories of such maturational changes remain elusive. Here, we aim to study how the brain engages in multiple tasks (working memory, reward processing, and inhibition) at the network-level and evaluate how effects of age across these tasks are related to each other. We characterise how the brain departs from its functional baseline architecture towards task-induced functional connectivity modulations using a novel measure called *task potency*, allowing direct comparison between tasks by defining sensitivity to one or multiple tasks. By applying this method in a sample of healthy participants (N = 218) aged 8–30 years, we demonstrate maturational changes in task-dependent functional co-activation over and above baseline connectivity maturation. Our results provide evidence for task-specific maturational windows with different cognitive systems probed by different tasks displaying specific age-range dependencies of strongest developmental change. Our results highlight the use of task potency for modelling developmental trajectories and the impact of differential maturation across tasks. This enables better characterisation of cognitive processes disrupted in neurodevelopmental disorders and may explain the increased level of heterogeneity observed in adolescent population studies.

## Highlights

• Task potency quantifies task-induced functional connectivity modulation from baseline.
• Task potency characterizes shared age effects across multiple tasks on connectivity.
• Task-modulation of edges is related across tasks.
• We detected task-specific maturational dynamics in developmental trajectories.

## 1 Introduction

Understanding human cognition in adolescent cohorts is invariably linked to understanding cognitive maturation and brain development. Multiple theories aimed at modelling development build on the seminal work of Jean Piaget to explain maturation of cognitive abilities (Mareschal, 2011). The interactive specialization theory states that cognitive functions interact in their maturation (Elman et al., 1996, Johnson, 2011). For instance, the maturation of working memory and processing speed is predictive of language maturation (Newbury et al., 2016).

Experimental evidence for the interactive specialization theory supports its notion that maturation is a combination of planned biological, experience-induced, and learning-induced changes (Astle et al., 2015, Buschkuehl et al., 2012). Neuroconstructivism further proposes that learning-induced maturation applies to the cellular-, brain network-, and cognitive function-levels. As cognitive functions would not mature independently, brain networks would also not mature independently (Newcombe, 2011, Westermann et al., 2007), i.e., developmental changes in reward processing will impact the development of inhibition, and would be reflected in neural correlates of this maturational interaction between neural networks. As an example, in maturation of memory, emergence of knowledge can be modelled from interactions between prior knowledge (Brod et al., 2013). To assess the idea of co-occurring development, it becomes necessary to investigate underlying common maturational processes between cognitive functions and their neural correlates.

Task-based fMRI has been instrumental in assessing hypotheses that relate brain development to such cognitive maturation. However, due to periods of rapid development during adolescence the use of a single task across a large age-range to characterise cognitive maturation remains practically challenging. Moreover, many studies examine changes in a single fMRI task with development, which limits the generalizability of possible conclusions. In this context, resting state fMRI has been put forward as a viable alternative as it can be administered across ages regardless of cognitive abilities. Although resting state fMRI allows investigation of the brain’s baseline functional architecture (Smith et al., 2009), and can predict task responses (Kelly et al., 2008, Mennes et al., 2010, Tavor et al., 2016), observed changes with age in the brain’s resting architecture might not be sufficient to explain maturation in cognitive performance. As studies indicate that task-related connectivity builds on the brain’s baseline functional architecture, it is clear that resting state connectivity does not capture all neural processes that are related to task performance (Kelly et al., 2008, Mennes et al., 2013a). Therefore, resting state-derived results provide insight into a different aspect of brain functioning yet cannot substitute task-based fMRI studies that aimed to link a specific cognitive function to specific brain areas (Stevens, 2016). Assessing the additional value of task-fMRI in understanding cognitive maturation requires dissociating age-related changes in the brain’s baseline architecture from age-related changes in task-induced neural modulations departing from that baseline. Ideally, this would incorporate multiple experimental tasks allowing to obtain insight into task- or function-specific versus -common patterns of maturation.

Relying on the availability of both resting-state fMRI and task-fMRI data we use a novel analytical approach to define task-modulated functional connectivity that enables us to look at common maturational effects across multiple tasks. Importantly, we index task-induced modulations independent of generic maturational changes in the brain’s baseline architecture. More specifically, we focus on so-called *task-potency*, an index that compares functional connectivity under task performance relative to the brain’s generic baseline functional architecture as measured using resting-state functional connectivity. This is based on the idea that engaging in a task causes modification of functional connectivity away from its baseline status (Kelly et al., 2008, Mennes et al., 2013a), in a way that allows prediction of the task modality (Geerligs et al., 2015, Tavor et al., 2016). This is enabled by the idea that resting state represents the landscape of cognitive states through fluctuation of large-scale networks (Shen et al., 2015, Shine et al., 2016, Smith et al., 2009) and allows to capture specificities of an individual (Mueller et al., 2013, Shen et al., 2017). As task potency is readily comparable across tasks we can investigate the existence of singular versus common maturational processes across cognitive functions, allowing us to investigate the idea of co-occurring development. We here demonstrate that characterisations on the basis of task potency give rise to interpretable differential developmental trajectories of different cognitive systems.

Such comparisons between tasks while incorporating resting state fMRI by means of the task potency measure offer great potential in the context of large-scale neuroimaging efforts that include multiple tasks acquired in large cohorts (e.g., NKI-RS (Nooner et al., 2012), the HCP Lifespan Project (Glasser et al., 2013), UK Biobank (Miller et al., 2016), and FCP-INDI (Mennes et al., 2013b)). Instead of analysing individual task responses independently, task potency focuses on integration across multiple tasks (Chauvin et al., 2017). Here, we used a local database providing three fMRI tasks (working memory, reward processing and inhibition) acquired alongside a resting-state scan in a large developmental cohort and assessed the impact of age on task-induced connectivity modulations. Specifically, we focussed on the relationship of age effects between tasks and their potentially common underlying maturational processes.

## 2 Methods

### 2.1 Participants

In the current analyses, we use MRI data from healthy control participants only (initial N = 385) of the NeuroIMAGE sample (Rhein et al., 2015) who each performed at least one of the following tasks during fMRI scanning: response inhibition (Stop Signal Task (STOP), (Logan et al., 1984, Rhein et al., 2015, van Rooij et al., 2015)), reward processing (REWARD, (Hoogman et al., 2011, Knutson et al., 2001, Rhein et al., 2015, von Rhein et al., 2015)), spatial working memory (WM, (van Ewijk et al., 2015, Klingberg et al., 2002, McNab et al., 2008, Rhein et al., 2015)). In addition, each participant completed a resting state (RS) fMRI session. fMRI acquisition parameters are shown in Table 1. All participants also completed an anatomical scan for registration purposes (T1-weigthed MPRAGE, TR = 2730 ms, TE = 2.95 ms, T1 = 1000 ms, flip angle = 7, matrix size = 256 × 256, FOV = 256 mm, 176 slices with 1 mm isotropic voxels).

**Table 1:** fMRI acquisitions parameters.

	RS	STOP	REWARD	WM
**Image acquisition parameters**				
General parameters	TE = 40 ms, FOV = 224 mm, 37 axial slices, flip angle = 80, matrix size = 64 × 64, in-plane resolution = 3.5 mm, slice thickness/gap = 3.0 mm/0.5 mm			
N volumes	>260	86 * 4 blocks	>300	107 * 4 blocks
TR (ms)	1960^b^	2340^b^	2340^b^	2340
N first volumes rejected^a^	5	4	5	3

**Participant characteristics**				
N used in final analyses	218	111	123	144
RMS-FD min-max	0.026–1.930	0.029–0.413	0.027–0.554	0.033–1.504
RMS-FD mean (std)	0.171 (0.224)	0.09 (0.074)	0.13 (0.099)	0.17 (0.23)
Age min–max	8.6–30.5	8.6–27	9.1–23.9	8.6–27
Age mean (std)	17 (3.5)	17.1 (3.5)	16.8 (3.2)	16.8 (3.2)
% female	54.1%	54.0%	57.8%	52.8%

a The number of initial volumes removed from further analyses varied to ensure comparability with earlier studies that used these data. Note that this variation will have very limited impact on the current analyses.

b some subjects were scanned with a different TR: RS – 1860 for 11 subjects; STOP – 2150 for 10; REWARD – 2150 for 10.

FMRI scans exhibiting limited brain coverage or excessive head motio n were excluded from further processing. Limited brain coverage was defined as having less than 97% overlap with the MNI152 standard brain after image registration. Applying this criterion excluded 47 subjects. In addition, we excluded from each task those participants who were among top 5% in terms of head motion as quantified by RMS-FD, the root mean square of the frame-wise displacement computed using MCFLIRT (Jenkinson et al., 2002). Applying these criteria resulted in the inclusion of data from 218 healthy controls, comprising 218 resting state acquisitions, 111 STOP acquisitions, 123 REWARD acquisitions, and 144 WM acquisitions. Participants ranged in age between 8.6 and 30.5 years; mean = 16.9; sd = 3.4; 54.1% were female. Further details are included in Table 1 and Supplementary Fig. 1.

### 2.2 fMRI preprocessing

All fMRI acquisitions were processed using tools from FSL 5.0.6. (FSL; http://www.fmrib.ox.ac.uk/fsl) (Jenkinson et al., 2012, Smith et al., 2004, Woolrich et al., 2009). We employed the following pipeline: removal of the first volumes to allow magnetization equilibration (Table 1), head movement correction by volume-realignment to the middle volume using MCFLIRT, global 4D mean intensity normalization, spatial filtering with a 6 mm FWHM Gaussian kernel. Subsequently we applied ICA-AROMA, an automated algorithm to detect head motion-related artefacts in single-subject fMRI data based on independent component analysis. ICA components identified as related to head motion were subtracted out of the data using fsl_regfilt (Pruim et al., 2015a, Pruim et al., 2015b). Finally, we regressed out mean signals from CSF and white matter, and applied a 0.01 Hz temporal high-pass filter.

For each participant, all acquisitions were registered to its high-resolution T1 image using Boundary-Based Registration (BBR) available in FSL FLIRT (Jenkinson and Smith, 2001, Jenkinson et al., 2002). All high-resolution T1 images were registered to MNI152 space using 12-dof linear registration available in FLIRT and further refined using non-linear registration available in FSL FNIRT (Anderson, 2007).

### 2.3 Region-of-interest analysis

For each functional imaging scan we defined connectivity matrices using regions defined in a hierarchical whole-brain functional atlas (van Oort et al., 2017). This atlas contains 185 non-overlapping regions and was defined through Instantaneous Correlation Parcelation (ICP) applied to resting state fMRI data of 100 participants of the Human Connectome Project (HCP; (Glasser et al., 2013)). In short, ICP aims to parcel larger regions into subregions based on signal homogeneity, where the optimal number of subregions is determined based on split-half reproducibility at the cohort level.

**Fig. 1:**
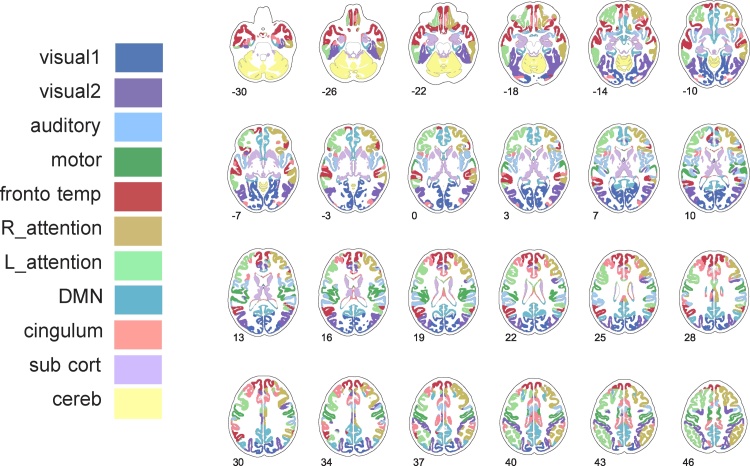
179 areas selected from an ICP-based parcellation of the human brain (van Oort et al., 2017). Each area is coloured in accordance to its overarching network. Eleven large-scale networks constitute the first level of the parcellation: visual1, visual2, auditory, motor, fronto-temporal (fronto temp), right and left attention (R_attention, L_attention, respectively), default mode (DMN), cingulum, sub-cortical (sub cort), cerebellum (cereb) networks. We used the 179 regions that are part of the sub-network scale parcellation to obtain functional fingerprints based on 179 × 179 correlation matrices.

Fig. 1 illustrates the hierarchical brain atlas, where areas were grouped into 11 higher-level networks: 9 resting state networks (visual1, visual2, motor, right attention, left attention, auditory, default mode network (DMN), fronto-temporal and striatum), and 2 anatomical structures (subcortical areas, and cerebellum). These higher-level networks respectively contained 19, 12, 22, 22, 18, 8, 18, 13, 7, 23, and 23 subregions.

All analyses were performed in each participant’s native space. To this end we transformed the atlas to each participant’s native space using the inverse of the anatomical to MNI152 non-linear warp, and the inverse of the linear transformation of the functional image to the participant’s high resolution anatomical image. Voxel-membership in brain parcels was established on the basis of majority overlap. Areas that were on average across our population over 50% outside of the brain were rejected from further analyses. This resulted in the rejection of one area in brainstem. For consistency, we removed the 5 other brainstem areas. As a result, we used 179 areas to compute connectivity matrices, as explained in Section 2.4.

### 2.4 Connectivity calculation

For each participant and each task (RS, WM, REWARD, STOP) we calculated 179 × 179 connectivity matrices, by cross-correlating the time series of all regions in our atlas. We obtained each region’s time series through multivariate spatial regression, using all 179 regions as regressors and each task’s preprocessed time series as dependent variable. The resulting regional time series were demeaned. For the WM and STOP task we temporally concatenated the time series of individual runs. Using these time series, we calculated 179 × 179 partial correlation matrices through inverting covariance matrices estimated by the Ledoit-Wolf normalization algorithm (Ledoit and Wolf, 2004) as implemented in nilearn (http://nilearn.github.io/). Finally, all pair-wise correlations were Fisher *r*-to-*Z* transformed.

**Fig. 2:**
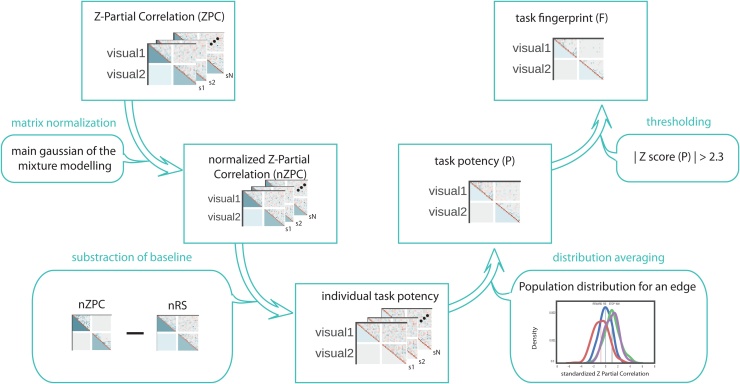
Task-potency pipeline. Using the brain parcellation shown in Fig. 1, we calculated 179 × 179 connectivity matrices for each individual in each task (WM, REWARD, STOP, RS). From the Fisher r-to-Z transformed partial correlation, we obtained task potency by first normalizing the task and rest connectivity and subsequently subtracting the rest from the task connectivity. Through population averaging and thresholding the resulting matrices we obtained a task potency fingerprint for each task (WM, REWARD, STOP).

To allow comparison of connectivity values between acquisitions, we normalized the connectivity values within each matrix to fit a Gaussian distribution (Fig. 2). Importantly, we were cautious not to affect the tails of the connectivity distributions as these represent the most interesting connectivity values. Therefore, we modelled the obtained connectivity values per task using a Gaussian-gamma mixture-model to obtain “mixture-model-corrected” Z-stat values (Feinberg et al., 2010, Llera et al., 2016). This model fits three curves to represent the data: a central Gaussian distribution representing the noise and two gamma distributions on each side of the central Gaussian that represent the signal as the tails of the data distribution. We used the main Gaussian, i.e., the one fitting the body of the distribution, to normalize our connectivity values with respect to its main distribution (i.e., noise), while not taking into account the extremes (i.e., signal). In practice, we applied the mixture modelling to the upper triangle values of each connectivity matrix and subsequently normalized the connectivity values by subtracting the mean and dividing by the standard deviation of the obtained central Gaussian model. As a result, the values within the normalized, *Z*-transformed partial correlation matrices are readily comparable across tasks (Feinberg et al., 2010).

To differentiate connectivity changes induced by task modulation from changes in the baseline architecture, we standardized each participant’s task-based connectivity matrix by the population average resting state matrix. This effectively allows interpretation of the task-based connectivity matrices in terms of their deviation from the resting state baseline connectivity. Accordingly, we can interpret the resulting values as ‘task potency’, referring to the magnitude of the task-induced connectivity modulation. We standardized each individual-level connection, i.e. entry in the correlation matrix, by subtracting its own individual-level connection value obtained during rest. As such, each task-based pair-wise correlation or edge quantifies how connectivity for that edge differed from that edge’s connectivity during the resting state. For each participant we obtained a standardized connectivity matrix for each of its task acquisitions, further referred to as task potency matrices. For each task, we finally created group-level task potency matrices by averaging across the participant-level matrices.

### 2.5 Task-based fingerprints

To focus on maturational change of connections that characterize a task’s functional fingerprint we selected – for each task – those edges that showed a relevant deviation from rest (see Fig. 2 right half). To this end we converted the group-level task potency matrices to *Z*-statistic matrices by subtracting the mean and dividing by the standard deviation calculated for each task matrix. For each task we then selected those edges with an absolute *Z*-statistic > = 2.3. This threshold was chosen to represent 2.3 standard deviation from the Gaussian noise of the baseline distribution, thereby respecting the logic of sparseness, i.e., strong connectivity modulations occur infrequently, and corresponding to a *p*-value of 0.01 for each end of the task-potency distribution. We refer to those selected edges as task-modulated edges and to the resulting matrices as *task-based fingerprints*. Here, we defined task-based fingerprints at the group-level by selecting edges in the task potency matrix as averaged across the population. Group fingerprints describe each task-potency architecture and can be used to address common connectivity modulations between tasks. Note that it is also possible to create fingerprints at the individual level, i.e., the individual task connectivity matrix adjusted for its individual resting state connectivity matrix. Individual potency fingerprints reflect individual variability in the task potency architecture and can be directly compared to its group-level equivalent. We did not investigate individual-level potency in this study.

### 2.6 Investigating effects of age

We investigated age-related effects on task potency based on the underlying idea that task connectivity modulations that are in common between tasks reflect underlying common mechanisms. Accordingly, we investigated age-related effects on the potency of single edges as well as on an average potency across subgroups of edges. For both analyses, we used least square fitting to investigate the linear change with age, thereby maximising the detection of maturational processes while minimizing the complexity of the model. We applied correction for multiple comparisons across the tested subgroups of edges by implementing FDR correction (*q <* 0.05).

**Fig. 3:**
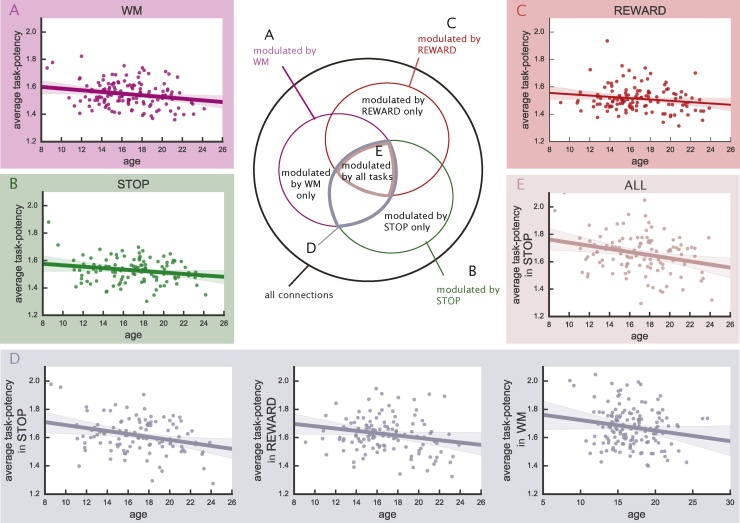
Effect of age on edges modulated by each task. Each graph illustrates the effect of age for corresponding edges indicated in the Venn-diagram. **A**: edges modulated during WM; **B**: edges modulated during STOP; **C**: edges modulated during REWARD; **D**: edges modulated by WM and STOP; **E**: edges modulated by all three tasks. All displayed effects, except for **C**, reached statistical significance at p < 0.05 after FDR correction. The age effect is calculated by linear regression of age against the average potency over the specified subset of edges. The average potency decreases significantly with age for edges selected in STOP and WM, in all tasks for edges shared by WM and STOP, and in STOP for edges shared by all three tasks.

The subgroups of edges we used were, for each task, 1) edges modulated by this task only, 2) edges modulated by this task and one of the two other tasks, 3) edges modulated by all three tasks. See the Venn-diagram in Fig. 3 for an overview of potential edge subgroups. We propose that similar changes with age will be observed across tasks in connections that they co-modulate. For example, if task potency in one task increases with age for an edge modulated by more than one task, we would expect to observe a similar increase with age in all other tasks modulating this edge.

The average potency across edges within each of the edge subgroups specified above reflects an average underlying mechanism, but potentially obscures effects that play at the single edge level. To gain insight into age effects at the level of single edges we compared the slope of the linear relationship between age and potency for each edge within the task-modulation fingerprint of two tasks. Specifically, we plot the slope of each edge in one task against the corresponding slope of that edge in the other task. We then fit an ellipsoid on the resulting scatter plot using least square fitting to quantify the relationship between the two displayed tasks. If the ellipsoid stretched around the *x* *=* *y* diagonal axis, it indicates a strong relationship between the two parameters, which in our case translates into the observation that connectivity modulation would mature similarly in both tasks. We conducted this analysis independently in edges shared by the two tasks or selected in only one task. To quantify the strength of the relationship we calculated the width/height ratio of the ellipsoid fit. The closer this ratio is to 1, the rounder the ellipsoid, and the weaker the relationship between the two tasks.

Finally, at the single edge level, we tested for second order changes with age, i.e. we tested whether the speed of the maturational changes varied as a function of age. We assumed that age effects would be stronger in younger than in older participants. To this end, we modelled a linear change over a short age window of 1 year including 7 participants from this window. When more than 7 participants were available within an age window we randomly selected 7. We moved this window across our entire population, each time removing the youngest subject of the window and considering a 1 year age span starting from the age of the subject immediately following in age. We extracted the absolute beta value of the linear regression for each window as a marker for the speed of change with age of the task-potency.

### 2.7 Disentangling baseline and task-modulation effects

In order to confirm that task-potency changes with age were not solely driven by changes with age in the baseline (i.e., resting-state derived) connectivity, we assessed whether age also impacted baseline connectivity and whether potential age effects on the baseline related to age effects on task-based connectivity modulations. To this end we also conducted our age-based analyses on the baseline connectivity, i.e. the normalized *Z*-partial correlation extracted from the resting state scan. We compared age effects obtained for baseline and for task modulation and evaluated whether both measures were related by correlating the baseline connectivity score with the task-potency across subjects. At the edge level, we defined the fingerprint of the baseline connectivity by selecting edges with |*Z*| > 2.3. We assessed the correspondence between age effects on shared selected connections in each task with age-related changes observed at baseline by least-square fitting an ellipsoid as done for the comparison between tasks.

## 3 Results

### 3.1 General effects of age on task-potency for selected edges

**Fig. 4:**
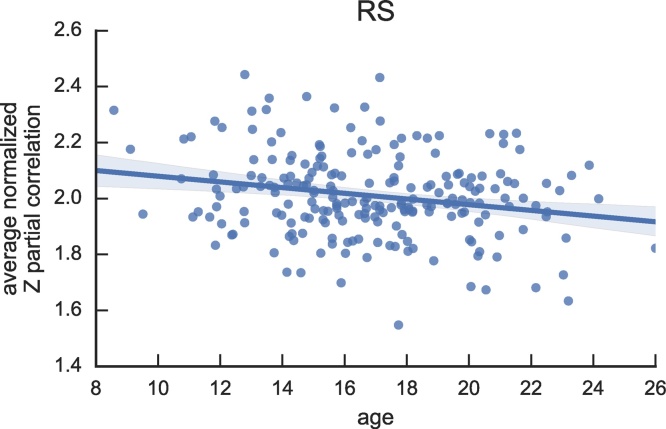
Effect of age on baseline, i.e., resting state connectivity. Age is linearly regressed against the average normalized Z partial correlation over edges selected in the resting state fingerprint. Connectivity decreases significantly with age at p < 0.05.

For both the STOP and WM task we observed that task-potency across edges modulated by each task decreased significantly with age. This suggests that in each of these tasks, as participants mature, their task-modulation and baseline fingerprint become more similar to each other (Fig. 3A and B). In addition, we observed a significant decrease with age in baseline connectivity (Fig. 4).

To confirm that the task-potency changes with age were not driven by changes with age in the baseline connectivity, we correlated the average potency observed under task modulation to the average connectivity in the baseline condition across the population. The average was computed independently for edges modulated by the STOP and the WM task. We observed no correlation between the resting connectivity and task potency for either task: *r*(STOP, REST) = 0.026; *r*(WM, REST) = −0.054. This suggests that the modulation of connectivity under task performance shows developmental changes that are independent of the maturational changes in baseline functional connectivity.

### 3.2 Effect of age on task-potency for common edges across tasks fingerprints

To investigate common underlying maturational mechanisms across tasks, we estimated the effect of age on average potency across selected edges modulated by multiple tasks. Edges modulated by both the STOP and the WM task showed a significant decrease in average potency as measured under STOP and WM modulation (Fig. 3D). To investigate whether the age effect is specific to tasks modulating theses edges, we also assessed the average potency of these edges in the REWARD task. While REWARD-related edges did not show a significant change with age in the average potency across selected edges, average REWARD-potency across selected edges shared by STOP and WM shows a significant decrease with age (Fig. 3D). The observation that edges sensitive to both WM and STOP also show an age effect under REWARD, although they are not sensitive to modulation by this task, suggests that maturation of task-modulation in one task can be transferred across tasks.

Such common effect of age could be due to the maturation of a subgroup of edges modulated by all three tasks. However, edges shared between all three tasks showed a decrease in average potency with age in the STOP task only (Fig. 3E). This result indicates that the age effect detected in edges modulated by STOP and WM is not dependent on shared selected edges with REWARD and supports the idea that task-connectivity modulation can be identical between tasks, even if the edges are not strongly modulated by each of the tasks. Additionally, the STOP task is the only task showing an age effect in edges shared by all tasks, which indicates existence of maturational processes attributed to a single task.

### 3.3 Visualization of areas related to shared edges between tasks showing an age effect

**Fig. 5:**
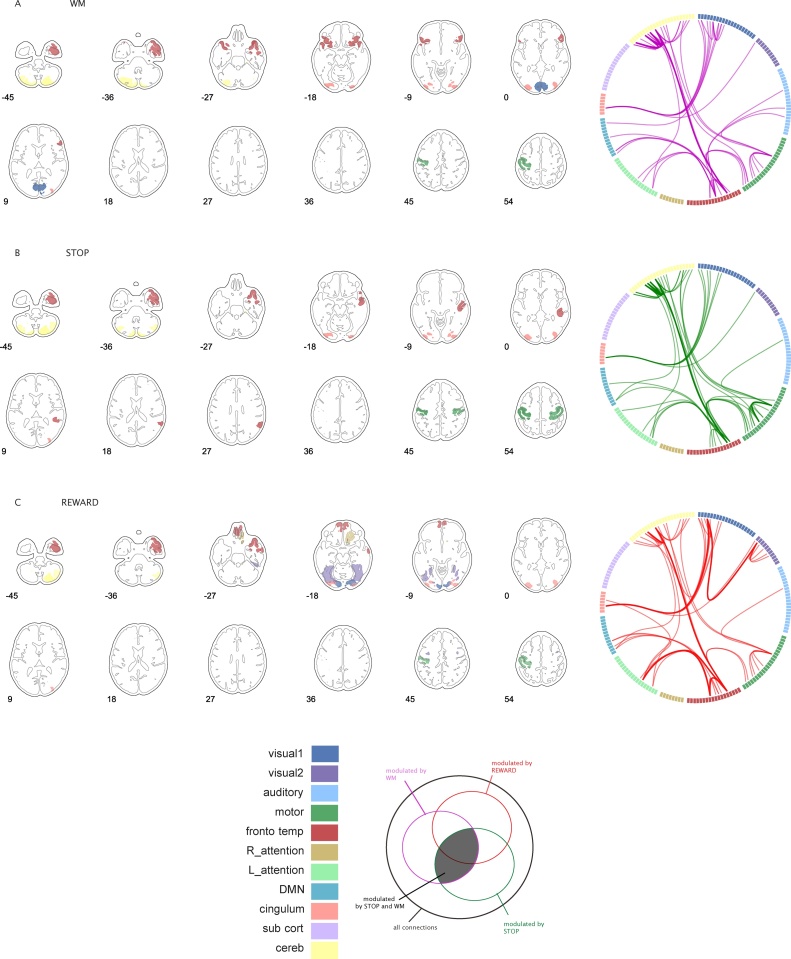
Top 5% areas showing the strongest linear age effects across edges modulated by STOP and WM tasks (darkest subgroup in the Venn-diagram, see also Fig. 3c). The linear age effect per edge, averaging and selection of the top 5% areas are done independently for each task and represented in A for WM, B for STOP, and C for REWARD. Circles represent the edges included in the top 5% area selection. Thicker edges in the circles represent those edges that formed a connection between two areas within the top selection.

Fig. 5 illustrates which areas are related to the edges exhibiting the top 5% strongest age effects across the edges modulated by both WM and STOP for which we observed age-related effects in all three tasks (see Fig. 3E). When comparing the edge representation in Fig. 5A, B, and C it is clear that, within the edges modulated by both STOP and WM, all three tasks displayed the strongest age effects between areas of visual1, fronto-temporal, cingulum, DMN, attention, and cerebellum networks. Of note, a subset of the displayed edges is not strongly modulated under REWARD, we have created separate visualizations of the strongest age effect for edges modulated by all tasks and for edges modulated under STOP and WM only. For these we refer to Supplementary Fig. 5a and b. Comparison of these separate figures enables differentiation of whether similarity across tasks is due to shared modulation. As the similarity between tasks generalized to both subsets of edges (Supplementary Fig. 5a and b), the current results suggest non-independence of age effects between these tasks, especially at the level of larger networks.

### 3.4 Effect of age on potency at the single edge level

**Fig. 6:**
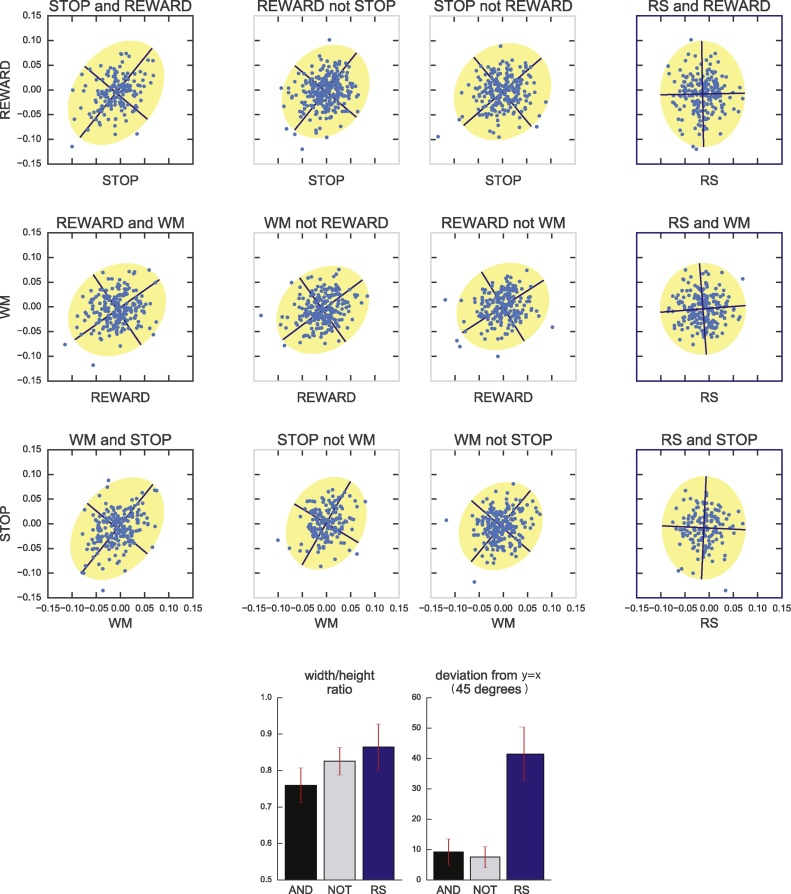
Relationship of age-effects between tasks for specific or common edges. A linear regression against age is computed for each edge in the task potency of each task. The beta parameters corresponding to the slope of the linear regression are extracted for each edge and related between two tasks. Edges displayed in the left column are edges selected in both tasks included in the plot, the two central columns display correspondence for edges selected in only one of the two tasks of the plot. The right column displays correspondence for edges selected in the baseline fingerprint (i.e. the resting state z partial correlation) versus one of the tasks. An ellipsoid is fit over the points in the scatter plot and two values are extracted from the ellipsoid: the deviation from 45° (i.e., x = y) for the main axis of the ellipsoid and the elongation of the ellipsoid (i.e. width divided by height). Bar plots on the bottom illustrate these parameters for each of the left (black), the two central (grey), and the right column (blue).

The age effect on the average potency of edge subgroups as presented in Section 3.2 does not provide fine-grained information about single edges. Here, we quantify the similarity of the age effect between tasks by estimating the age effect for each single edge and subsequently comparing between tasks. To this end, we computed the effect of age for all selected edges in each task. Using edges related to a pair of tasks, we conducted two comparisons of their age effects: 1) between edges shared by that pair of tasks, and 2) between edges modulated by only one task within the pair. We assessed this relationship by fitting ellipsoids to a scatter plot of the data. When edges showed related age effects between tasks we expected to observe an ellipsoid elongated along the diagonal where x = y. As shown in Fig. 6, first column, we observed an ellipsoid around the diagonal axis for edges shared between each pair of tasks (average 9.75 ° deviation from x = y axis with an average width/height ratio of the ellipsoid of 0.75).

For edges only selected in one of the two tasks, we expected that correspondence between the age effects would be less strong, resulting in rounder ellipsoids. As evident in the two middle columns of Fig. 6 we indeed observed rounder ellipsoids with a width/height ratio closer to 1, yet with a conserved orientation towards the x = y diagonal. This result supports the idea that task connectivity modulations share maturational processes that also impact modulation from tasks that are involved in a different task fingerprint.

To add further verification that the age effect on task modulation (Fig. 3) was not related to the age effect on baseline connectivity (Fig. 4), we compared the baseline and the task modulation age effects at the edge level. We expected that the ellipsoid would show a reduced or absent orientation towards the diagonal as a marker of un-related maturational processes. Using edges selected in the task modulation fingerprint and in the baseline fingerprint, we displayed the effect of age on the resting state connectivity against the effect of age on the task potency for each edge and observed that the resulting ellipsoid fit showed no specific orientation and a strong elongation over the task modulation axis (see Fig. 6 right column). This indicates that age effects observed for task potency and baseline connectivity were not related, suggesting that different maturational processes impact task modulation and resting connectivity.

### 3.5 Developmental dynamics at the individual edge level

**Fig. 7:**
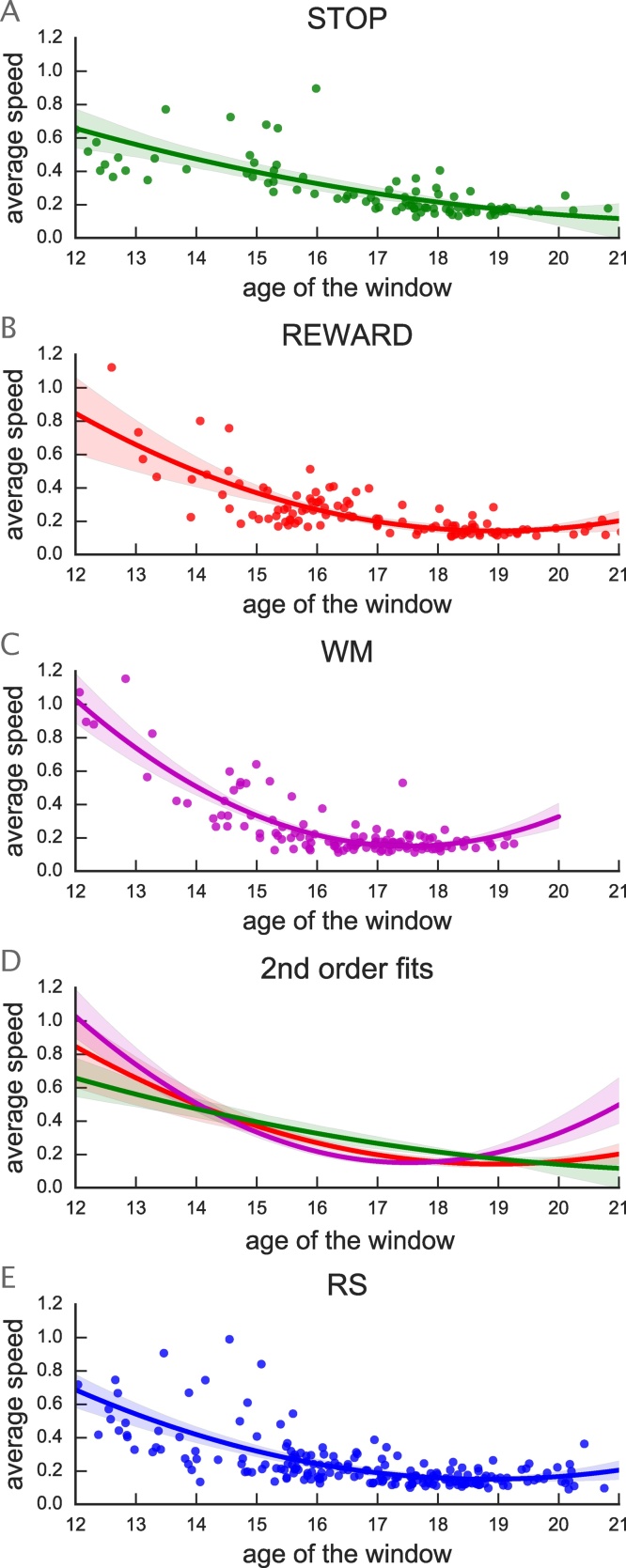
Average speed of change with age of task potency for STOP (A), REWARD (B), and WM (C). Each plot illustrates the absolute beta-parameters for each window in the sliding-average calculation. For each task we fit a 2nd order polynomial to model the rate of change across development. Graph D overlays each task’s 2nd order fit to allow comparison between tasks. Finally, E illustrates the rate of change for RS. Note that due to different input to the regression models, the amplitude for RS should not be directly compared to the amplitude for the other plots.

To assess the dynamics of the observed age effect across the age range of our population we modelled the linear change with age using a sliding window approach. Fig. 7, illustrates the maturational dynamics as indexed by the average slope of the effect of age across the selected edges per task. All tasks (Fig. 7A–C) showed a non-linear trajectory across their maturational window. Compared to STOP, both REWARD and WM exhibited stronger age-related effects before age 15 (Fig. 7B & C). In contrast (Fig. 7A), the STOP task exhibited overall slower and more linear maturational dynamics continuing until age 18, suggesting more gradual maturational effects across our age range. This difference between tasks in the timing of maturational changes suggests that brain activity related to each task has a specific maturational window (Fig. 7D). Combined with the finding that maturation is related between tasks at the edge level (Fig. 6), the observation that the maturational dynamics have different timing is consistent with the idea that maturation in one task can influence maturation of another task. Here, we can speculate that faster developmental changes of WM and REWARD-related circuitry until age 15 potentially influences the continued STOP task maturation actually requiring smaller (but prolonged) developmental changes.

The resting state connectivity also exhibited more gradual dynamics, with the strongest maturational changes occurring before age 17 (Fig. 7E). Importantly, the difference in amplitude of change between tasks and RS cannot be interpreted as the input data are of different nature, i.e., task potency (adjusted for rest) versus functional connectivity.

## 4 Discussion

We introduced task potency as a sensitive feature to study brain involvement in cognitive tasks across development. The feature is reflecting connectivity modulations under performance of a task relative to functional connectivity observed during a resting state. To study development, task potency enables dissociating changes with age in the brain’s baseline functional connectivity architecture from changes with age in functional connectivity as elicited across multiple tasks.

We observed task-specific maturation independent of age-related effects on the baseline (i.e., resting state) connectivity patterns (Figs. A, B, and). For STOP and WM (Fig. 3D), we observed that task potency decreased with age. At the same time, we observed that between-region resting state functional connectivity also decreased with age, thereby replicating previous studies (Stevens et al., 2009). Importantly, we showed that the age-related effects on task potency were not related to the resting state maturation, both at the level of task-specific edges, as well as at the single edge level (Fig. 6).

Decreasing task potency with age indicates that the task-based and resting state connectivity architectures converge with age, allowing reduced switching costs to transition from a baseline state towards a task state. The convergence between task-based and resting state connectivity exhibited task-specificity, i.e., the REWARD task showed a different developmental trajectory relative to WM and STOP. This result replicates earlier findings from a meta-analysis where reward tasks showed significantly different task-dependant connectivity compared to task-independent resting state connectivity in adults (Kellermann et al., 2013). Note that the absence of a task-independent maturational effect, does not exclude the possibility of common maturational processes that are shared between select tasks, e.g., observed that STOP and WM showed a similar age effect as their average potency in the subset of edges shared by these two tasks decreased with age (Fig. 3D). This decrease of task potency shared by the two tasks supports the idea of a shared underlying neural maturational process, located in a subset of edges modulated by both tasks. Moreover, this common maturational process did extend to REWARD, as the average potency of edges shared by the STOP and WM tasks also exhibited decreasing potency with age in REWARD. Such co-maturation could be converging towards or supported by an architecture of flexible multi-task hubs as observed by Cole and colleagues (Cole et al., 2013).

By comparing common modulations across tasks, task potency enabled to define edges involved in the maturation of multiple cognitive functions. This allows developing new hypotheses to study how cognitive functions relate to one another. For example, if two related cognitive functions mature over two different time windows, the cognitive function that matures earlier will impact the maturation of the second one. We observed support for such hypothesis by investigating the difference in maturational dynamics between tasks. Specifically, we observed that REWARD and WM exhibited the strongest maturational changes at earlier ages compared to the STOP task (Fig. 7). However, without a larger observation window, we cannot distinguish whether the STOP task simply displays a more gradual change across development or whether its strongest maturational changes happened in earlier developmental phases. Longitudinal data across a larger age window, would be required to allow investigating whether the bigger individual age effects in WM and REWARD require smaller maturational changes in the STOP task. The difference in timing of maturation between reward processing and inhibition relates to the idea that motivation and executive control interact during maturation through alterations in the communication between striatum and prefrontal cortex (PFC) (Somerville and Casey, 2010). In the context of detecting salient environmental cues during adolescence, striatum, involved in early temporal coding of reward, would trigger bottom-up maturation of the connection between striatum and PFC. In contrast, top-down connections from PFC to striatal areas, reflecting cognitive control, mature only afterwards. Corroborating this idea, we showed connectivity modulation between areas typically involved in executive functioning (Chung et al., 2014, Diamond, 2013) exhibited the strongest age-related effects (Fig. 5). This result is in accordance the fact that executive functioning, being strongly associated to PFC functioning, is one of the cognitive functions that is thought to mature late, not reaching completion until early adulthood (Carriedo et al., 2016). Accordingly, through comparison of appropriate tasks and age-windows, task potency could be used, for instance, to predict inhibition-specific maturational changes related to PFC maturation. Yet, in this context, we highlight that our approach assumes that our normalisation approach allows isolating task-driven connectivity changes. However, the separation of exogenously-driven modulations from low-frequency fluctuations found in both resting state and task-related fMRI timeseries remains a matter of active empirical examination. While, the current results fit this presumption, it will require additional research of the neurophysiological basis of connectivity and its complex relationship to cross-correlated BOLD signal dynamics throughout the brain to know whether this assumption is fully supported.

Linking changes in connectivity to behavioural changes would provide more insight into how potentiation of edges matters for the maturation of cognitive functioning. Supplementary Fig. 8 illustrates the maturational dynamics for the most typical behavioural parameter in each task. Similar to the maturational dynamics observed for task potency (Fig. 7), the behavioural parameter for the STOP task (i.e., SSRT) did exhibit a more gradual change across the age window of our sample. In comparison, the behavioural parameters for REWARD (reward-related speeding) and WM (error rate) exhibited faster developmental changes in earlier ages. Our results corroborate behavioural studies providing evidence for maturation of cognitive abilities across our age range. For spatial working memory, a strong increase in the number of remembered items occurs between 11 and 15 years old (Conklin et al., 2007), while response inhibition exhibits a gradual increase in performance until adult-level performance is reached around age 15 (Luna et al., 2004, van den Wildenberg and van der Molen, 2004). In addition, studies showing that maturation of reward processing can influence maturation of inhibition provide evidence for underlying common neural correlates of both cognitive process (Geier and Luna, 2012, Geier et al., 2010). However, it is clear that we cannot assume that these different behavioural metrics integrate the same biological underlying processes. A reaction time and an error rate will reflect a different integration of the processes involved in proper task performance. To address the relativeness of task at the behavioural level, common mental processes across task need to be defined (Poldrack et al., 2011).

A common concern for developmental studies that make use of functional MRI data is the impact of head motion (Satterthwaite et al., 2012). During preprocessing we have used ICA-AROMA to mitigate effects of participant head motion on the collected data (Pruim et al., 2015a, Pruim et al., 2015b; for an independent evaluation of ICA-AROMA see e.g., Ciric et al., 2017). However, as some younger subjects showed highest head motion (see Supplementary material Fig. 2), and given that it has been shown that head motion is heritable (Engelhardt et al., 2017), it is possible that head motion might relate to underlying biological features of interest and will accordingly exhibit maturational changes. To account for this potential interaction effect and to further validate our results, we replicated all results using a linear model including both age and head motion. Results can be found in Supplementary Figs. 3, 4, 6, and 7. Overall, results were comparable between the different models, with limited changes in some relationships not reaching significance anymore, while others did reach significance when including head motion in the model. These changes can be due to the use of a more complex model, and to amplification of the age effect when movement-related variance is modelled out, helping some age effects to reach significance.

Observing neural mechanisms of maturation that affect multiple tasks and their associated cognitive functions provides support for the interactive specialization theory (Elman et al., 1996, Johnson, 2011) and neuroconstructivism (Newcombe, 2011, Westermann et al., 2007), two related developmental theories stating that cognitive functions interact in their maturation. Our results corroborate earlier experimental evidence supporting the notion that maturation is a combination of planned biological, experience-induced, and learning-induced changes (Astle et al., 2015, Buschkuehl et al., 2012). Neuroconstructivism in particular proposes that learning-induced maturation applies to the cellular-, brain network-, and cognitive function-levels. As cognitive functions would not mature independently, brain networks would also not mature independently (Newcombe, 2011, Westermann et al., 2007), i.e., developmental changes in reward processing will impact the development of inhibition, and would be reflected in neural correlates of this maturational interaction between neural networks. We observed an age effect on REWARD-related potency in edges that showed strong task involvement and a strong age effect in the STOP and WM tasks (Fig. 3c). The observation that these edges were not strongly involved in REWARD processing suggests that theses edges are trained, i.e. matured, by STOP and WM performance. This training then impacts these edges’ connectivity as observed under REWARD processing. We could not differentiate whether the age-related effects on the edges shared by STOP and WM tasks represent a common maturational mechanism or maturation of an independent cognitive function involved in all three tasks that would be evolving on its own (McNab et al., 2008). Longitudinal investigations would further enable to better understand variability in maturation between individuals and the specificity of task-related maturational processes. In this context, longitudinal measurement of resting state is of key importance to compare local age effects relative to local variability in resting state that is influenced by individual characteristics, experimental manipulations, or environmental factors. Accordingly, we encourage to obtain resting state data in the same session as the task scans (see also Chauvin et al., 2017).

Future investigations could examine why a reduction in task-induced connectivity modulations is a marker of brain maturity, possibly distinguishing effects of changes at the neurophysiological level from changes in the brain’s response to a task. In connectivity studies, some authors interpret a reduction with age of resting state connectivity as a reduced need for energy for a network to function and a more efficient integration of information (Stevens et al., 2009). This interpretation can also apply to task potency: a reduced switch from the baseline when engaging in a task can reflect a more efficient integration of information. This would support the idea that executive function performance is associated to higher flexibility in connectivity, allowing more frequent switching from one connectivity state to another (Nomi et al., 2017). A lower task potency request facilitates such flexibility by making switching between rest and task less costly. We can hypothesise that a reduced need of modulation to reach the requested connectivity state would be beneficial for a better performance by reducing the cost of involvement in executive tasks. This hypothesis would need further validation. Investigating this hypothesis in the context of ADHD could provide such validation as it has been theorized that individuals with ADHD have difficulty in energizing their brain activity (Sergeant, 2005). We could investigate whether impairment of ADHD participants in executive functioning is linked to higher task potency levels displayed during tasks. If so, we could predict under what cognitive load or when ADHD participants would experience cognitive fatigue as the demand for task-induced modulations becomes too high. In general, investigation of cognitive impairment in developmental disorders such as ADHD is intrinsically linked to understanding deviant task-related modulations related to differences in the baseline brain architecture due to age effects and/or clinical representations. The task potency framework is well suited to enable researchers to detect and understand differences linked to cognitive performance in various domains of impairment, thereby tapping into both cortical and subcortical networks.

In conclusion, understanding how human cognition matures, requires defining not only functional connectivity changes in the baseline, but also changes in functional connectivity that is modulated by tasks (Stevens, 2016). Our study shows that task potency defined as the difference in connectivity modulation between rest and task is a promising neural correlate to study cognitive development.

## Conflict of Interest

None.

## References

[bib0005] Anderson M.L. (2007). The massive redeployment hypothesis and the functional topography of the brain. Philos. Psychol..

[bib0010] Astle D.E., Barnes J.J., Baker K., Colclough G.L., Woolrich M.W. (2015). Cognitive training enhances intrinsic brain connectivity in childhood. J. Neurosci..

[bib0015] Brod G., Werkle-Bergner M., Shing Y.L. (2013). The influence of prior knowledge on memory: a developmental cognitive neuroscience perspective. Front. Behav. Neurosci..

[bib0020] Buschkuehl M., Jaeggi S.M., Jonides J. (2012). Neuronal effects following working memory training. Dev. Cogn. Neurosci..

[bib0025] Carriedo N., Corral A., Montoro P.R., Herrero L., Rucián M. (2016). Development of the updating executive function: from 7-year-olds to young adults. Dev. Psychol..

[bib0030] Chauvin R., Mennes M., Buitelaar J., Beckmann C. (2017). Reverse inference via connectivity fingerprinting: task sensitivity, task specificity, and task potency. bioRxiv.

[bib0035] Chung H.J., Weyandt L.L., Swentosky A., Goldstein S., Naglieri J.A. (2014). The physiology of executive functioning. Handbook of Executive Functioning.

[bib0040] Ciric R., Wolf D.H., Power J.D., Roalf D.R., Baum G.L., Ruparel K., Shinohara R.T., Elliott M.A., Eickhoff S.B., Davatzikos C. (2017). Benchmarking of participant-level confound regression strategies for the control of motion artifact in studies of functional connectivity. Neuroimage.

[bib0045] Cole M.W., Reynolds J.R., Power J.D., Repovs G., Anticevic A., Braver T.S. (2013). Multi-task connectivity reveals flexible hubs for adaptive task control. Nat. Neurosci..

[bib0050] Conklin H.M., Luciana M., Hooper C.J., Yarger R.S. (2007). Working memory performance in typically developing children and adolescents: behavioral evidence of protracted frontal lobe development. Dev. Neuropsychol..

[bib0055] Diamond A. (2013). Executive functions. Annu. Rev. Psychol..

[bib0060] Elman J.L., Bates E., Johnson M.H., Karmiloff-Smith A., Parisi D., Plunkett K. (1996). Rethinking Innateness.

[bib0065] Engelhardt L.E., Roe M.A., Juranek J., DeMaster D., Harden K.P., Tucker-Drob E.M., Church J.A. (2017). Children’s head motion during fMRI tasks is heritable and stable over time. Dev. Cogn. Neurosci..

[bib0070] Feinberg D.A., Moeller S., Smith S.M., Auerbach E., Ramanna S., Glasser M.F., Miller K.L., Ugurbil K., Yacoub E. (2010). Multiplexed echo planar imaging for sub-second whole brain FMRI and fast diffusion imaging. PLoS One.

[bib0075] Geerligs L., Rubinov M., Cam-CAN, Henson R.N. (2015). State and trait components of functional connectivity: individual differences vary with mental state. J. Neurosci..

[bib0080] Geier C.F., Luna B. (2012). Developmental effects of incentives on response inhibition. Child Dev..

[bib0085] Geier C.F., Terwilliger R., Teslovich T., Velanova K., Luna B. (2010). Immaturities in reward processing and its influence on inhibitory control in adolescence. Cereb. Cortex.

[bib0090] Glasser M.F., Sotiropoulos S.N., Wilson J.A., Coalson T.S., Fischl B., Andersson J.L., Xu J., Jbabdi S., Webster M., Polimeni J.R. (2013). The minimal preprocessing pipelines for the human connectome project. Neuroimage.

[bib0095] Hoogman M., Aarts E., Zwiers M., Slaats-Willemse D., Naber M., Onnink M., Cools R., Kan C., Buitelaar J., Franke B. (2011). Nitric oxide synthase genotype modulation of impulsivity and ventral striatal activity in adult ADHD patients and healthy comparison subjects. Am. J. Psychiatry.

[bib0100] Jenkinson M., Smith S. (2001). A global optimisation method for robust affine registration of brain images. Med. Image Anal..

[bib0105] Jenkinson M., Bannister P., Brady M., Smith S. (2002). Improved optimization for the robust and accurate linear registration and motion correction of brain images. Neuroimage.

[bib0110] Jenkinson M., Beckmann C.F., Behrens T.E.J., Woolrich M.W., Smith S.M. (2012). FSL. Neuroimage.

[bib0115] Johnson M.H. (2011). Interactive Specialization: a domain-general framework for human functional brain development?. Dev. Cogn. Neurosci..

[bib0120] Kellermann T.S., Caspers S., Fox P.T., Zilles K., Roski C., Laird A.R., Turetsky B.I., Eickhoff S.B. (2013). Task- and resting-state functional connectivity of brain regions related to affection and susceptible to concurrent cognitive demand. Neuroimage.

[bib0125] Kelly A.M.C., Uddin L.Q., Biswal B.B., Castellanos F.X., Milham M.P. (2008). Competition between functional brain networks mediates behavioral variability. Neuroimage.

[bib0130] Klingberg T., Forssberg H., Westerberg H. (2002). Training of working memory in children with ADHD. J. Clin. Exp. Neuropsychol..

[bib0135] Knutson B., Fong G.W., Adams C.M., Varner J.L., Hommer D. (2001). Dissociation of reward anticipation and outcome with event-related fMRI. Neuroreport.

[bib0140] Ledoit O., Wolf M. (2004). A well-conditioned estimator for large-dimensional covariance matrices. J. Multivar. Anal..

[bib0145] Llera, A., Vidaurre, D., Pruim, R.H.R., Beckmann, C.F., 2016. Variational Mixture Models with Gamma or inverse-Gamma components. ArXiv160707573 Stat.

[bib0150] Logan G.D., Cowan W.B., Davis K.A. (1984). On the ability to inhibit simple and choice reaction time responses: a model and a method. J. Exp. Psychol. Hum. Percept. Perform..

[bib0155] Luna B., Garver K.E., Urban T.A., Lazar N.A., Sweeney J.A. (2004). Maturation of cognitive processes from late childhood to adulthood. Child Dev..

[bib0160] Mareschal D. (2011). From NEOconstructivism to NEUROconstructivism. Child Dev. Perspect..

[bib0165] McNab F., Leroux G., Strand F., Thorell L., Bergman S., Klingberg T. (2008). Common and unique components of inhibition and working memory: an fMRI, within-subjects investigation. Neuropsychologia.

[bib0170] Mennes M., Kelly C., Zuo X.-N., Di Martino A., Biswal B.B., Castellanos F.X., Milham M.P. (2010). Inter-individual differences in resting-state functional connectivity predict task-induced BOLD activity. Neuroimage.

[bib0175] Mennes M., Kelly C., Colcombe S., Castellanos F.X., Milham M.P. (2013). The extrinsic and intrinsic functional architectures of the human brain are not equivalent. Cereb. Cortex.

[bib0180] Mennes M., Biswal B., Castellanos F.X., Milham M.P. (2013). Making data sharing work: the FCP/INDI experience. Neuroimage.

[bib0185] Miller K.L., Alfaro-Almagro F., Bangerter N.K., Thomas D.L., Yacoub E., Xu J., Bartsch A.J., Jbabdi S., Sotiropoulos S.N., Andersson J.L.R. (2016). Multimodal population brain imaging in the UK Biobank prospective epidemiological study. Nat. Neurosci..

[bib0190] Mueller S., Wang D., Fox M.D., Yeo B.T.T., Sepulcre J., Sabuncu M.R., Shafee R., Lu J., Liu H. (2013). Individual variability in functional connectivity architecture of the human brain. Neuron.

[bib0195] Newbury J., Klee T., Stokes S.F., Moran C. (2016). Interrelationships between working memory, processing speed, and language development in the age range 2–4 years. J. Speech Lang. Hear. Res..

[bib0200] Newcombe N.S. (2011). What is neoconstructivism?. Child Dev. Perspect..

[bib0205] Nomi J.S., Vij S.G., Dajani D.R., Steimke R., Damaraju E., Rachakonda S. (2017). Chronnectomic patterns and neural flexibility underlie executive function. Neuroimage.

[bib0210] Nooner K.B., Colcombe S.J., Tobe R.H., Mennes M., Benedict M.M., Moreno A.L., Panek L.J., Brown S., Zavitz S.T., Li Q. (2012). The NKI-Rockland sample: a model for accelerating the pace of discovery science in psychiatry. Front. Neurosci..

[bib0215] Poldrack R.A., Kittur A., Kalar D., Miller E., Seppa C., Gil Y., Parker D.S., Sabb F.W., Bilder R.M. (2011). The cognitive atlas: toward a knowledge foundation for cognitive neuroscience. Front. Neuroinf..

[bib0220] Pruim R.H.R., Mennes M., Buitelaar J.K., Beckmann C.F. (2015). Evaluation of ICA-AROMA and alternative strategies for motion artifact removal in resting state fMRI. Neuroimage.

[bib0225] Pruim R.H.R., Mennes M., van Rooij D., Llera A., Buitelaar J.K., Beckmann C.F. (2015). ICA-AROMA: a robust ICA-based strategy for removing motion artifacts from fMRI data. Neuroimage.

[bib0230] Rhein, von D., Mennes M., Ewijk H., van, Groenman A.P., Zwiers M.P., Oosterlaan J., Heslenfeld D., Franke B., Hoekstra P.J., Faraone S.V. (2015). The NeuroIMAGE study: a prospective phenotypic, cognitive, genetic and MRI study in children with attention-deficit/hyperactivity disorder. Design and descriptives. Eur. Child Adolesc. Psychiatry.

[bib0235] Satterthwaite T.D., Wolf D.H., Loughead J., Ruparel K., Elliott M.A., Hakonarson H., Gur R.C., Gur R.E. (2012). Impact of in-scanner head motion on multiple measures of functional connectivity: relevance for studies of neurodevelopment in youth. Neuroimage.

[bib0240] Sergeant J.A. (2005). Modeling attention-deficit/hyperactivity disorder: a critical appraisal of the cognitive-energetic model. Biol. Psychiatry.

[bib0245] Shen K., Hutchison R.M., Bezgin G., Everling S., McIntosh A.R. (2015). Network structure shapes spontaneous functional connectivity dynamics. J. Neurosci..

[bib0250] Shen X., Finn E.S., Scheinost D., Rosenberg M.D., Chun M.M., Papademetris X., Constable R.T. (2017). Using connectome-based predictive modeling to predict individual behavior from brain connectivity. Nat. Protoc..

[bib0255] Shine J.M., Bissett P.G., Bell P.T., Koyejo O., Balsters J.H., Gorgolewski K.J., Moodie C.A., Poldrack R.A. (2016). The dynamics of functional brain networks: integrated network states during cognitive task performance. Neuron.

[bib0260] Smith S.M., Jenkinson M., Woolrich M.W., Beckmann C.F., Behrens T.E.J., Johansen-Berg H., Bannister P.R., De Luca M., Drobnjak I., Flitney D.E. (2004). Advances in functional and structural MR image analysis and implementation as FSL. Neuroimage.

[bib0265] Smith S.M., Fox P.T., Miller K.L., Glahn D.C., Fox P.M., Mackay C.E., Filippini N., Watkins K.E., Toro R., Laird A.R. (2009). Correspondence of the brain’s functional architecture during activation and rest. Proc. Natl. Acad. Sci. U. S. A..

[bib0270] Somerville L.H., Casey B. (2010). Developmental neurobiology of cognitive control and motivational systems. Curr. Opin. Neurobiol..

[bib0275] Stevens M.C., Pearlson G.D., Calhoun V.D. (2009). Changes in the interaction of resting-state neural networks from adolescence to adulthood. Hum. Brain Mapp..

[bib0280] Stevens M.C. (2016). The contributions of resting state and task-based functional connectivity studies to our understanding of adolescent brain network maturation. Neurosci. Biobehav. Rev..

[bib0285] Tavor I., Jones O.P., Mars R.B., Smith S.M., Behrens T.E., Jbabdi S. (2016). Task-free MRI predicts individual differences in brain activity during task performance. Science.

[bib0290] van Ewijk H., Weeda W.D., Heslenfeld D.J., Luman M., Hartman C.A., Hoekstra P.J., Faraone S.V., Franke B., Buitelaar J.K., Oosterlaan J. (2015). Neural correlates of visuospatial working memory in attention-deficit/hyperactivity disorder and healthy controls. Psychiatry Res. Neuroimaging.

[bib0295] van Oort E.S.B., Mennes M., Navarro Schröder T., Kumar V.J., Zaragoza Jimenez N.I., Grodd W., Doeller C.F., Beckmann C.F. (2017). Functional parcellation using time courses of instantaneous connectivity. Neuroimage.

[bib0300] van Rooij D., Hartman C.A., Mennes M., Oosterlaan J., Franke B., Rommelse N., Heslenfeld D., Faraone S.V., Buitelaar J.K., Hoekstra P.J. (2015). Altered neural connectivity during response inhibition in adolescents with attention-deficit/hyperactivity disorder and their unaffected siblings. Neuroimage Clin..

[bib0305] van den Wildenberg W.P.M., van der Molen M.W. (2004). Developmental trends in simple and selective inhibition of compatible and incompatible responses. J. Exp. Child Psychol..

[bib0310] von Rhein D., Cools R., Zwiers M.P., van der Schaaf M., Franke B., Luman M., Oosterlaan J., Heslenfeld D.J., Hoekstra P.J., Hartman C.A. (2015). Increased neural responses to reward in adolescents and young adults with attention-deficit/hyperactivity disorder and their unaffected siblings. J. Am. Acad. Child Adolesc. Psychiatry.

[bib0315] Westermann G., Mareschal D., Johnson M.H., Sirois S., Spratling M.W., Thomas M.S.C. (2007). Neuroconstructivism. Dev. Sci..

[bib0320] Woolrich M.W., Jbabdi S., Patenaude B., Chappell M., Makni S., Behrens T., Beckmann C., Jenkinson M., Smith S.M. (2009). Bayesian analysis of neuroimaging data in FSL. Neuroimage.

