# Supplementary material for: Assessing age-dependent multi-task functional co-activation changes using measures of task-potency
Source: Dev Cogn Neurosci. 2017 Dec 5;33:5–16. doi: 10.1016/j.dcn.2017.11.011 (PMC6206256; doi:10.1016/j.dcn.2017.11.011)
Supplement: Supplementary file 1 [file mmc1.pdf]

Supplementary Material for Chauvin et al.: Assessing age-dependent multi-task functional co-activation changes using measures of task-potency

1. Description of task-fMRI paradigms

1.1. Stop signal task (STOP)

A visual version of the stop signal task (Logan et al., 1984; Rhein et al., 2015; van Rooij et al., 2015) was used to measure response inhibition during fMRI acquisition. In this task, participants had to respond as quickly as possible to a go-stimulus by left or right button press, unless shortly after presentation it was followed by a stop signal, in which case they were to withhold their response (25% of trials). The task consisted of two practice blocks and four test blocks, each consisting of 60 trials. For further details of the task and its acquisition parameters we refer to van Rooij et al., 2015.

1.2. Monetary reward processing task (REWARD)

A modified version of the MID task (Hoogman et al., 2011; Knutson et al., 2001; Rhein et al., 2015; von Rhein et al., 2015) was used to measure reward processing during fMRI acquisition. Participants were asked to respond as quickly as possible to a target by pressing a button. Prior to this target, a cue indicated the possibility to gain a reward after a button press within a given time window. Every trial ended with a feedback screen informing about the outcome of the current trial. Depending on the participants' performance, the response window for a correct response was adapted in the next trial resulting in an expected hit rate of 33%. The experiment lasted 12 minutes and a total of € 5 could be gained. For further details of the task and its acquisition parameters we refer to von Rhein et al., 2015.

1.3. Spatial working memory (WM)

The spatial span task used to measure spatial working memory is an adapted version of a task developed by Klingberg and colleagues (van Ewijk et al., 2015; Klingberg et al., 2002; McNab et al., 2008; Rhein et al., 2015). Two trial types (baseline and working memory) and two memory loads (low and high) were implemented in the task. Each trial consisted of a sequence of either three or six yellow circles (low and high memory load, respectively), displayed on a 4×4 grid for 500 ms each, with a 500 ms inter-stimulus interval in between. Subsequently, during a 2000 ms response window, a probe consisting of a number with a question mark was presented in one of the 16 locations. During working memory trials, participants were asked to remember the spatial location and temporal order of the presentation of cues, and indicate with a 'yes' or 'no' response (left or right button, respectively) whether the location of the probe had been stimulated before, at the indicated temporal position. During baseline trials, red circles followed by the probe (always the number 8) were presented sequentially in the four corners of the grid in a predictive manner, and participants were required to pay attention but not to try to remember the sequence, and always had to press the 'no' button. During both conditions, feedback was presented after the response in the form of a green or red coloured bar below the probe (for correct and incorrect responses, respectively), for the remainder of the response window. The task was administered in four blocks of 24 trials each (presented in fixed random order), with a short break in between blocks to motivate participants and to avoid fatigue effects, with a total task duration of approximately 16 min. For further details of the task and its acquisition parameters we refer to van Ewijk et al., 2015.

2. Supplementary Results

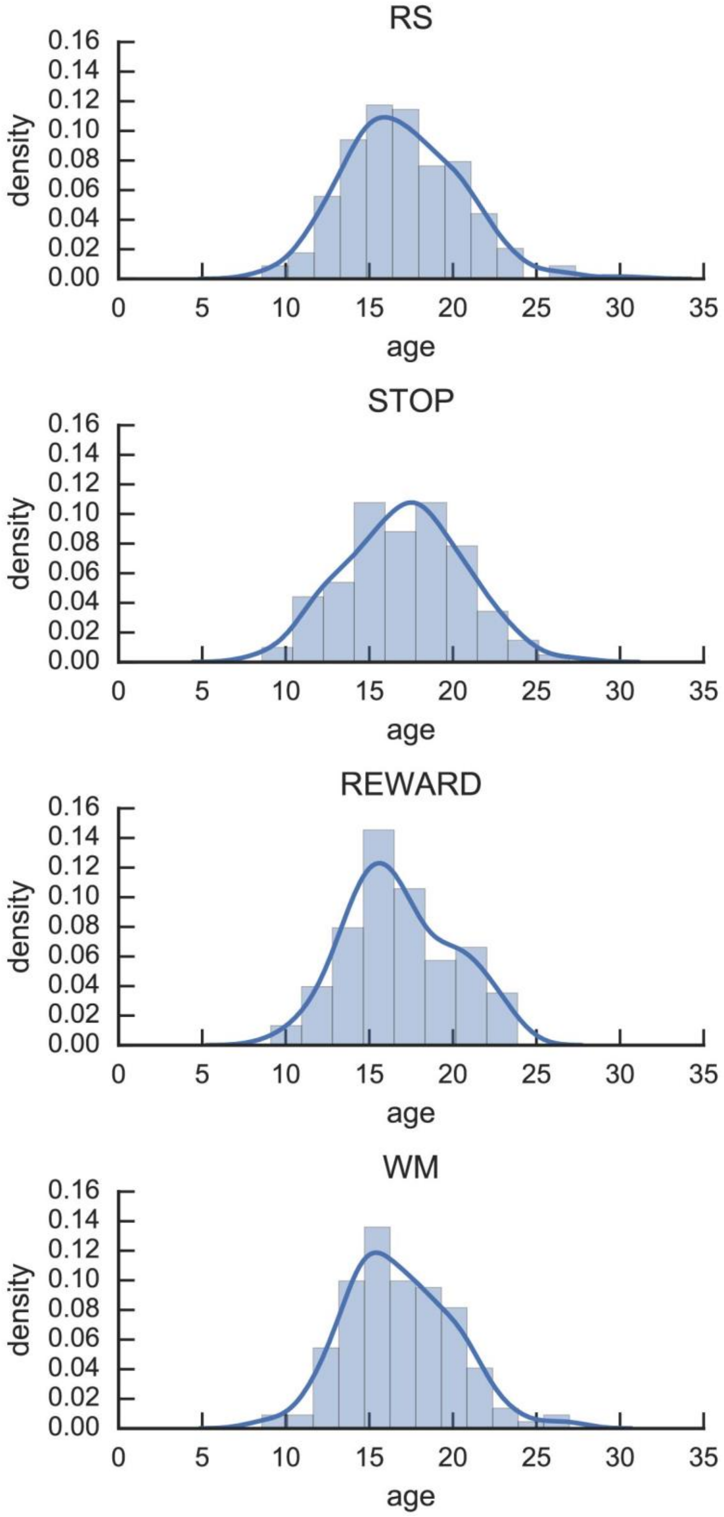

Supplementary Figure 1: distribution of participant age for each of the tasks

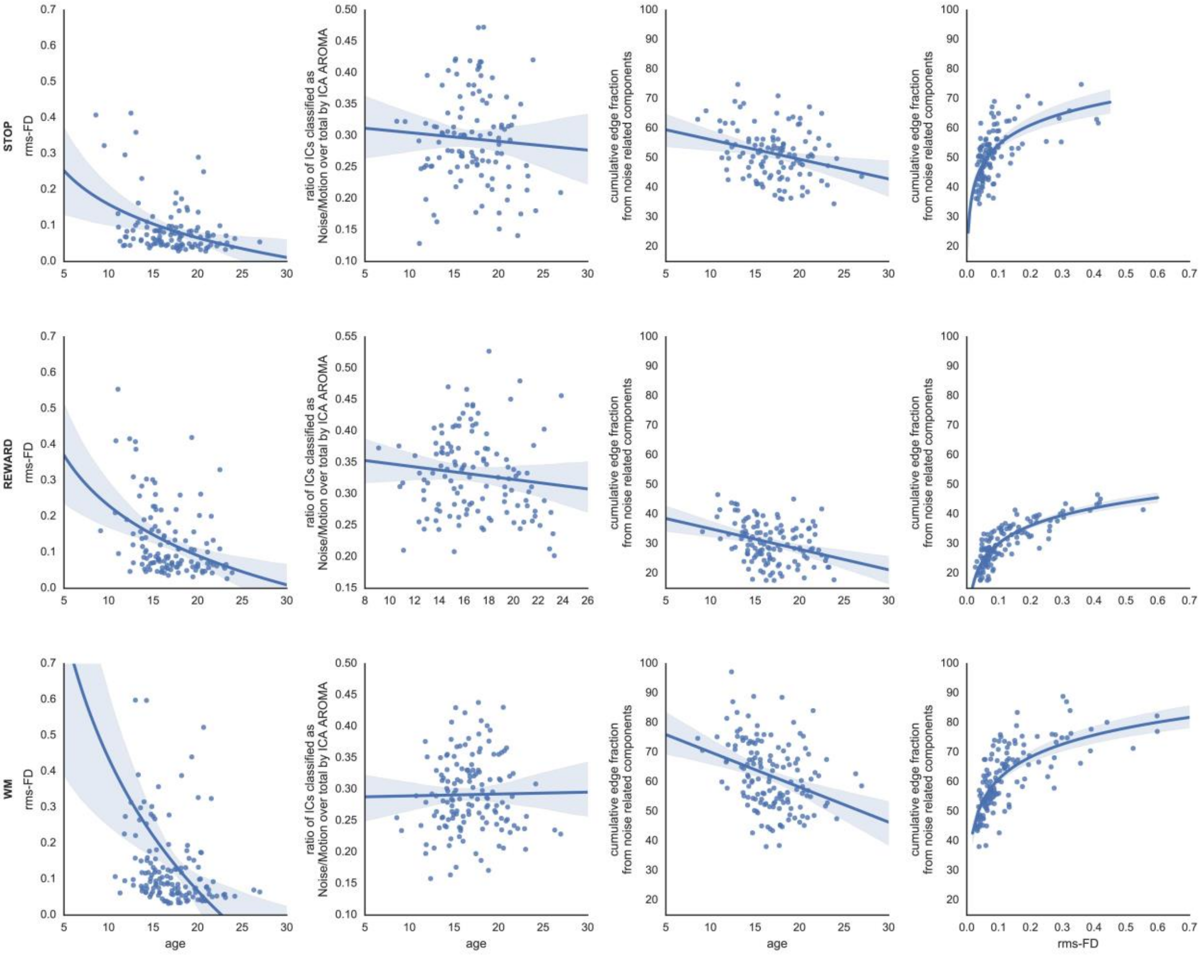

Supplementary Figure 2: Relationship between age and head motion-related metrics. The first column illustrates for each task (row) the relationship between age and rms-FD (Jenkinson et al., 2002). While age did not strongly correlate with head motion, it is clear that some younger participants displayed more extreme displacements. Column two illustrates the ratio between the number of components selected for correction by ICA-AROMA and the total number of components resulting from the ICA decomposition. This ratio is not related to age, illustrating that across participants there was no age-related bias in the number of components removed from the data. However, the selected components loaded more onto one of the parameters use to select ICs in ICA AROMA (edge fraction), showing that removed components more strongly represented head motion in younger participants. It is clear that this edge fraction is directly associated to head motion (column 4), supporting the notion that we do remove head motion-related noise and not signal of interest.

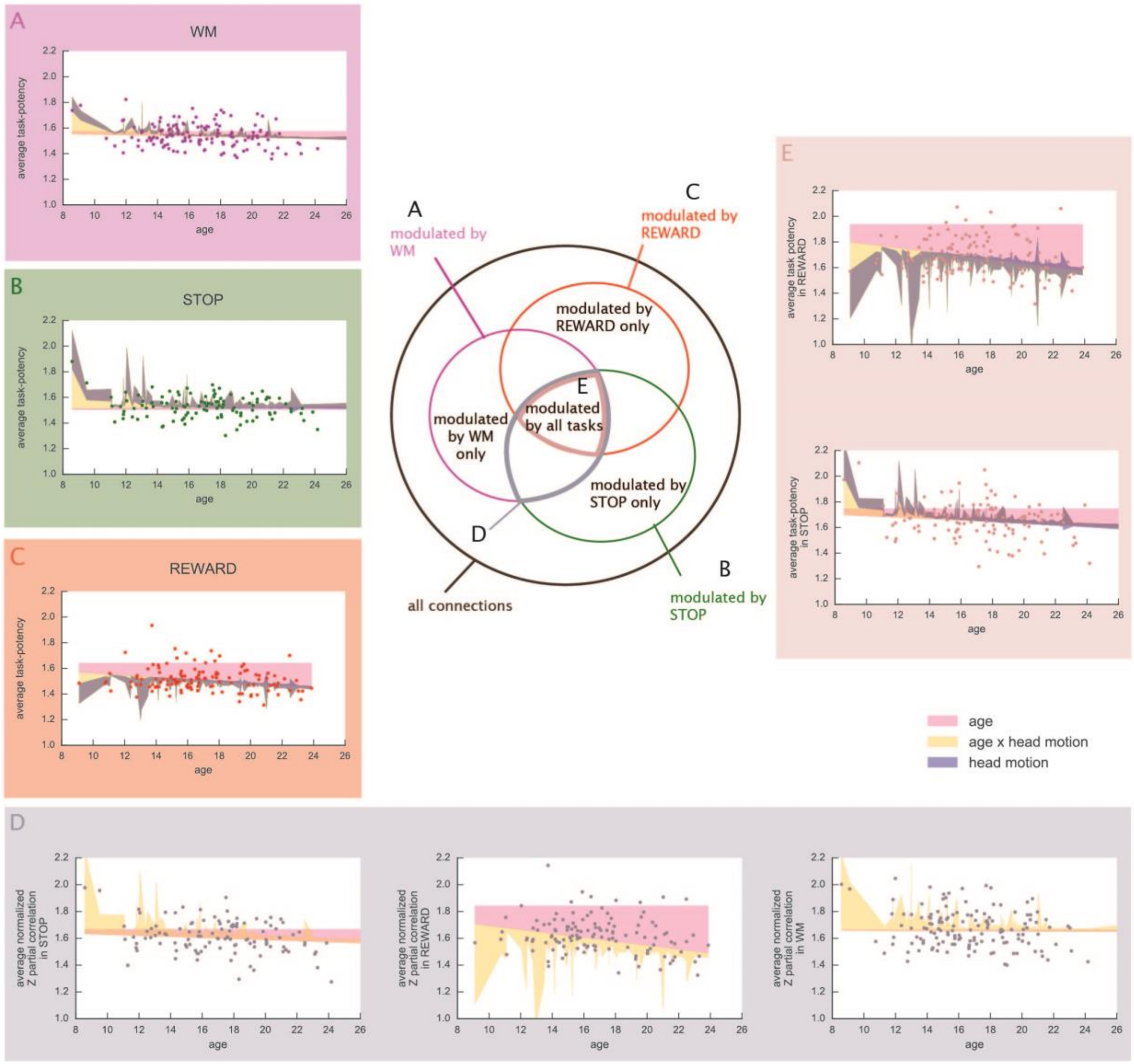

Supplementary Figure 3: Effects of age, age and head motion interaction, and head motion only on edges modulated by each task. Each graph corresponds to the analysis of the age effect on the average potency for corresponding edges indicated in the Venn-diagram. **A**: edges modulated during WM; **B**: edges modulated during STOP; **C**: edges modulated during REWARD; **D**: edges modulated by WM and STOP; **E**: edges modulated by all three tasks.

Each graph illustrates the additional effects of age only, age and head motion interaction, and head motion only from the following linear model: average task potency (P) = $\beta_0 + \beta_1$*age + β_2*(ageXhead motion) + β_3* head motion, where (ageXhead motion) is the interaction term of age and head motion, and β_i are the parameters. The dots correspond to the real value, the pink area corresponds to the change in value explained between 'P = β_0 ' and 'P = $\beta_0 + \beta_1$*age'. The orange area corresponds to the change in value explained between 'P = $\beta_0 + \beta_1$*age' and 'P = $\beta_0 + \beta_1$*age + β_2*(ageXhead motion)'. The blue area corresponds to the change in value explained between 'P = $\beta_0 + \beta_1$*age + β_2*(ageXhead motion)' and 'P = β_0 + β_1*age + β_2*(ageXhead motion) + β_3* head motion'.

Compared to figure 3 in the main text, supplementary figure 3 shows that some significant effects of age are lost possibly due to the loss of degrees of freedom due to using a higher-order model (see A, age effect on the average potency of edges sensitive to WM is not significant and see D, the age effect on edges modulated by all tasks is lost for STOP). In addition, some variance in task potency can be linked to head motion and can thus be modelled out, revealing an underlying age effect in edges sensitive to the REWARD task (see E), or modulated by all tasks in REWARD (see D first graph). However, even if some head motion can be interacting with the age effect, it can't be simply modelled out, as this interaction is significant in several cases, supporting the idea of a common underlying biology. See B and C (and supplementary table 2), when head motion shows a significant effect while age loses its significant effect compared to results in figure 3, the F-test across the age effect and the age by head motion interaction versus the head motion effect is significant. Modelling head motion without conserving the interaction would thus result in false negative age effects.

**Edges selection**	**Average potency**	**Factor**	**Coef**	**T score**	**p-value**	**F-test p-value**
A: Modulated by STOP		**Head motion**	0.4498	2.889	0.005	**0.00491**
		age	0.0008	0.283	0.778	
		**ageXhm**	-0.0260	-2.062	0.042	
B: Modulated by WM		Head motion	0.2136	1.608	0.110	0.1104
		age	-0.0024	-0.838	0.403	
		ageXhm	-0.0096	-0.986	0.326	
E: Modulated by REWARD		Head motion	-0.2827	-1.388	0.168	0.1702
		**age**	-0.0079	-2.215	0.029	
		ageXhm	0.0200	1.433	0.154	
C: Modulated by STOP and WM	In STOP	**Head motion**	0.4508	2.097	0.038	**0.039**
		age	-0.0039	-1.011	0.314	
		ageXhm	-0.0236	-1.357	0.178	
	In REWARD	**Head motion**	-0.6911	-2.462	0.015	**0.015**
		**age**	-0.0147	-2.984	0.003	
		**ageXhm**	0.0561	2.924	0.004	
	In WM	**Head motion**	0.4148	2.041	0.043	**0.044**
		age	-0.0005	-0.107	0.915	
		ageXhm	-0.0200	-1.336	0.184	
D: Modulated by all tasks	In REWARD	**Head motion**	-0.6922	-2.031	0.045	**0.045**
		**age**	-0.0151	-2.519	0.013	
		**ageXhm**	0.0479	2.056	0.042	
	In STOP	Head motion	0.4184	1.620	0.108	0.108
		age	-0.0056	-1.203	0.232	
		ageXhm	-0.0236	-1.128	0.262	

Supplementary Table 2: multifactor linear regression parameters linked to the regression plots shown in supplementary figure 3, the F-test corresponds to the analysis of age + ageXhead motion against head motion.

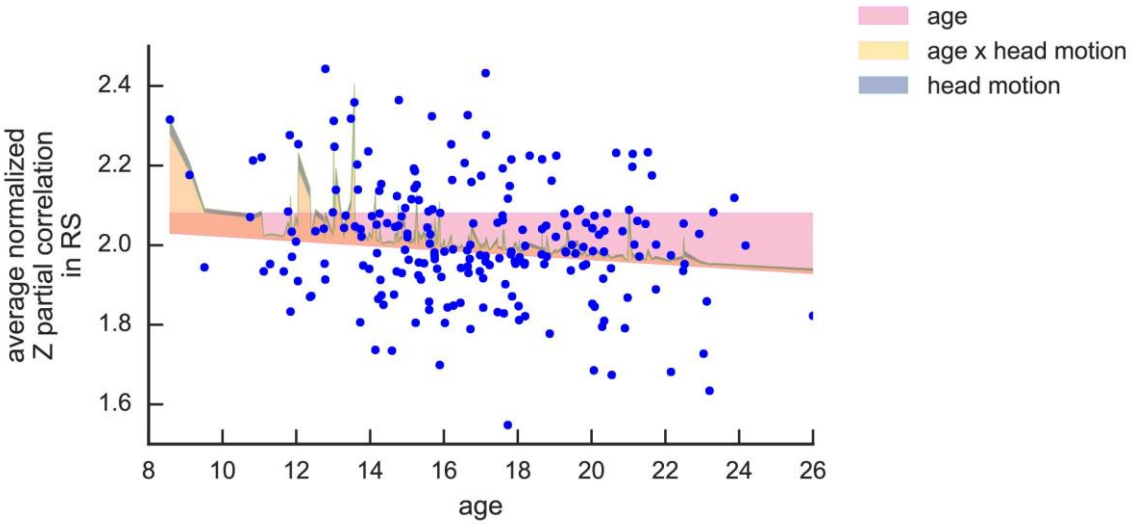

Supplementary Figure 4: Effect of age only, age and head motion interaction, and head motion only on resting state (RS) connectivity. The figure displays the additional effects of age only, age and head motion interaction, and head motion from the following linear model: average normalized Z partial correlation (P) = $\beta_0 + \beta_1$*age + β_2*(ageXhead motion) + β_3* head motion, where (ageXhead motion) is the interaction term of age and head motion, and β_i are the parameters. The dots correspond to the real value, the pink area corresponds to the change in value explained between 'P = β_0 ' and 'P = $\beta_0 + \beta_1$*age'. The orange area corresponds to the change in value explained between 'P = $\beta_0 + \beta_1$*age' and 'P = $\beta_0 + \beta_1$*age + β_2*(ageXhead motion)'. The blue area corresponds to the change in value explained between 'P = $\beta_0 + \beta_1$*age + β_2*(ageXhead motion)' and 'P = $\beta_0 + \beta_1$*age + β_2*(ageXhead motion) + β_3* head motion'. None of the parameters reach significance ($p<0.05$).

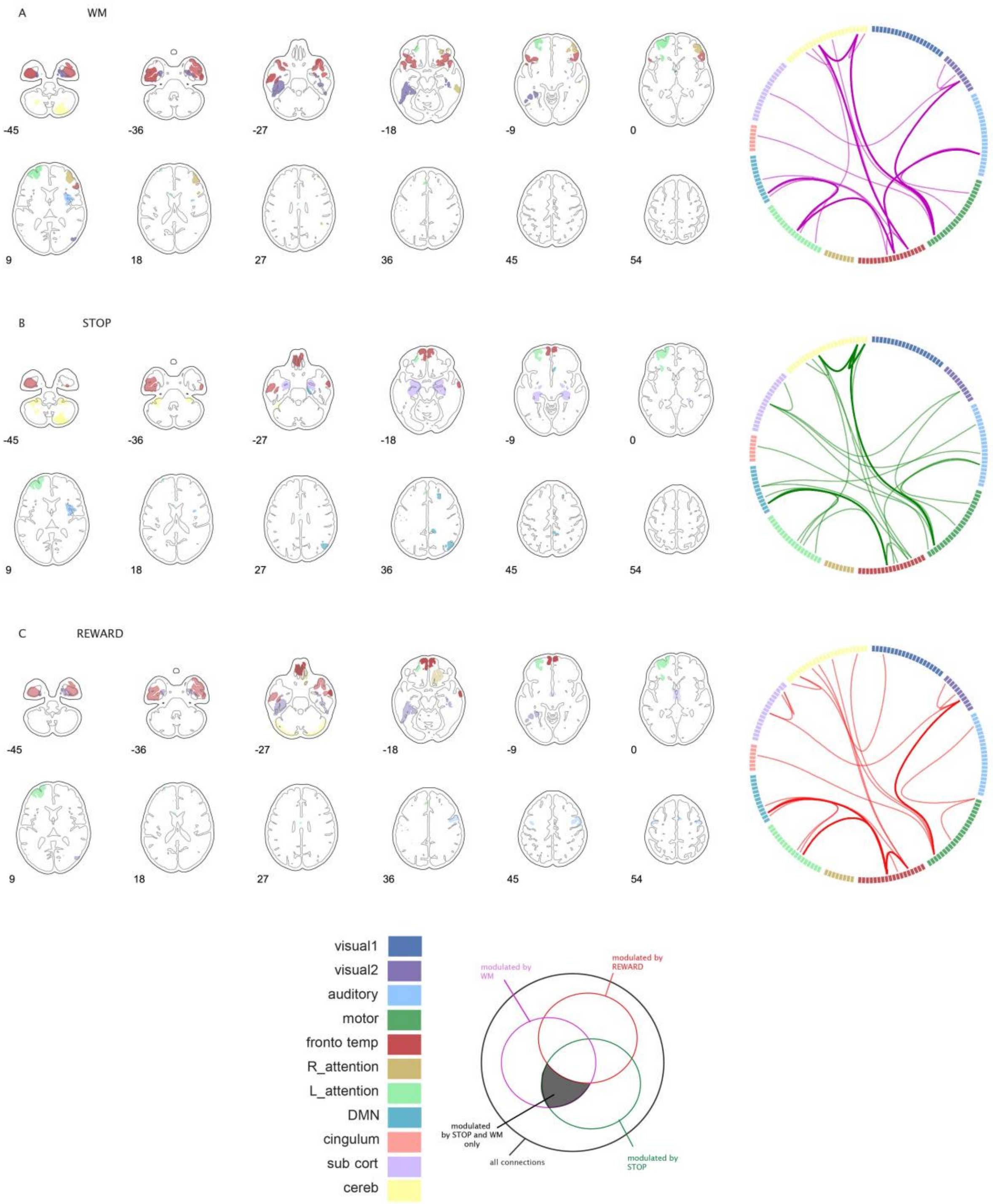

Supplementary Figure 5a: Top 5% areas showing the strongest linear age effects on average across edges modulated by STOP and WM only (darkest subgroup in the venn-diagram). The linear age effect per edge, average, and selection of the top 5% areas are done independently for each task and represented in A for the WM task, B for the STOP task, and C for the REWARD task. Circles represent the edges selected only in STOP and WM tasks of the top 5% areas selection. Thicker edges in the circle are edges that connect two areas within the top selection.

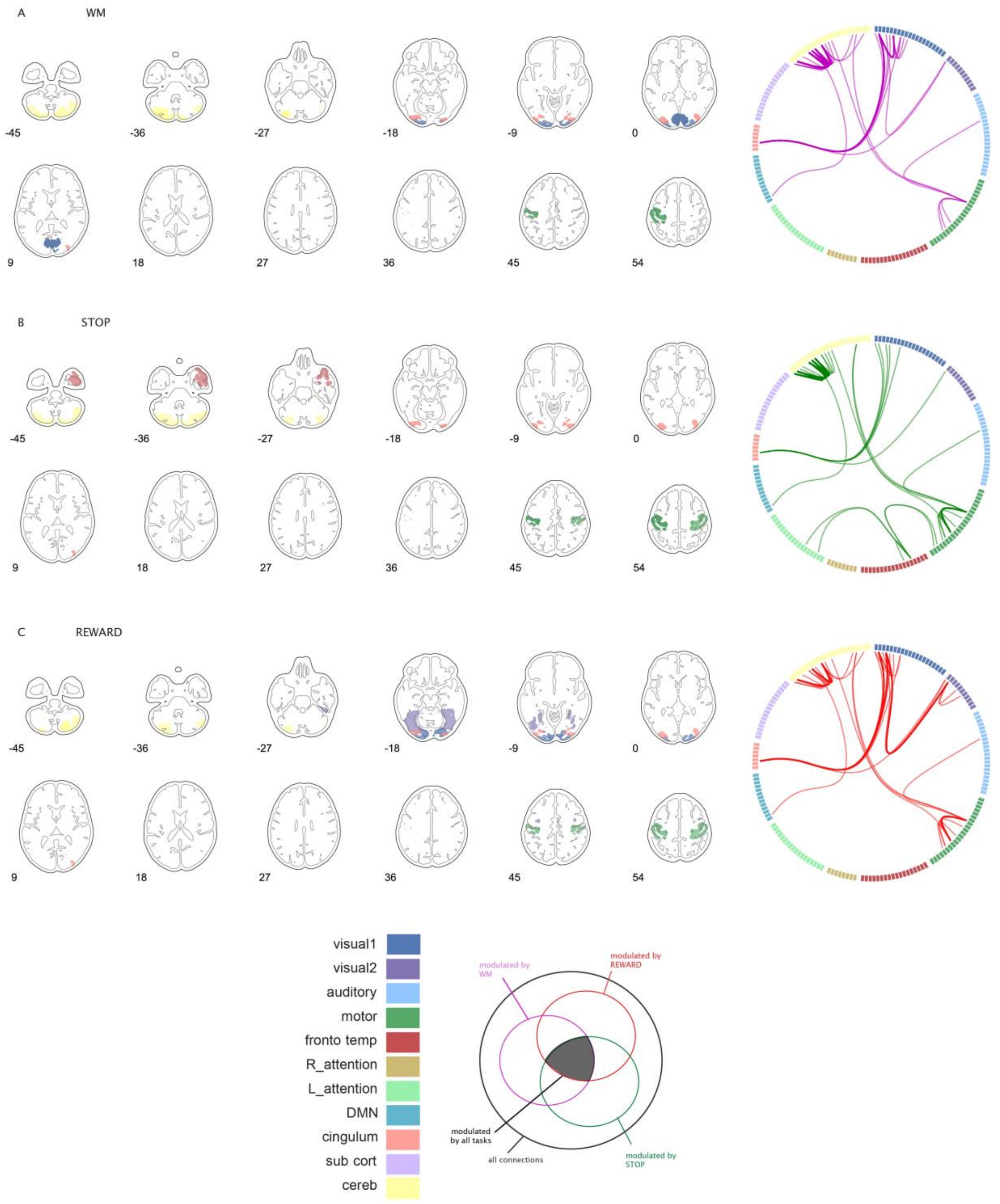

Supplementary Figure 5b: Top 5% areas showing the strongest linear age effects on average across edges modulated by all three tasks (darkest subgroup in the venn-diagram). The linear age effect per edge, average, and selection of the top 5% areas are done independently for each task and represented in A for the WM task, B for the STOP task, and C for the REWARD task. Circles represent the edges selected only in STOP and WM tasks of the top 5% areas selection. Thicker edges in the circle are edges that connect two areas within the top selection.

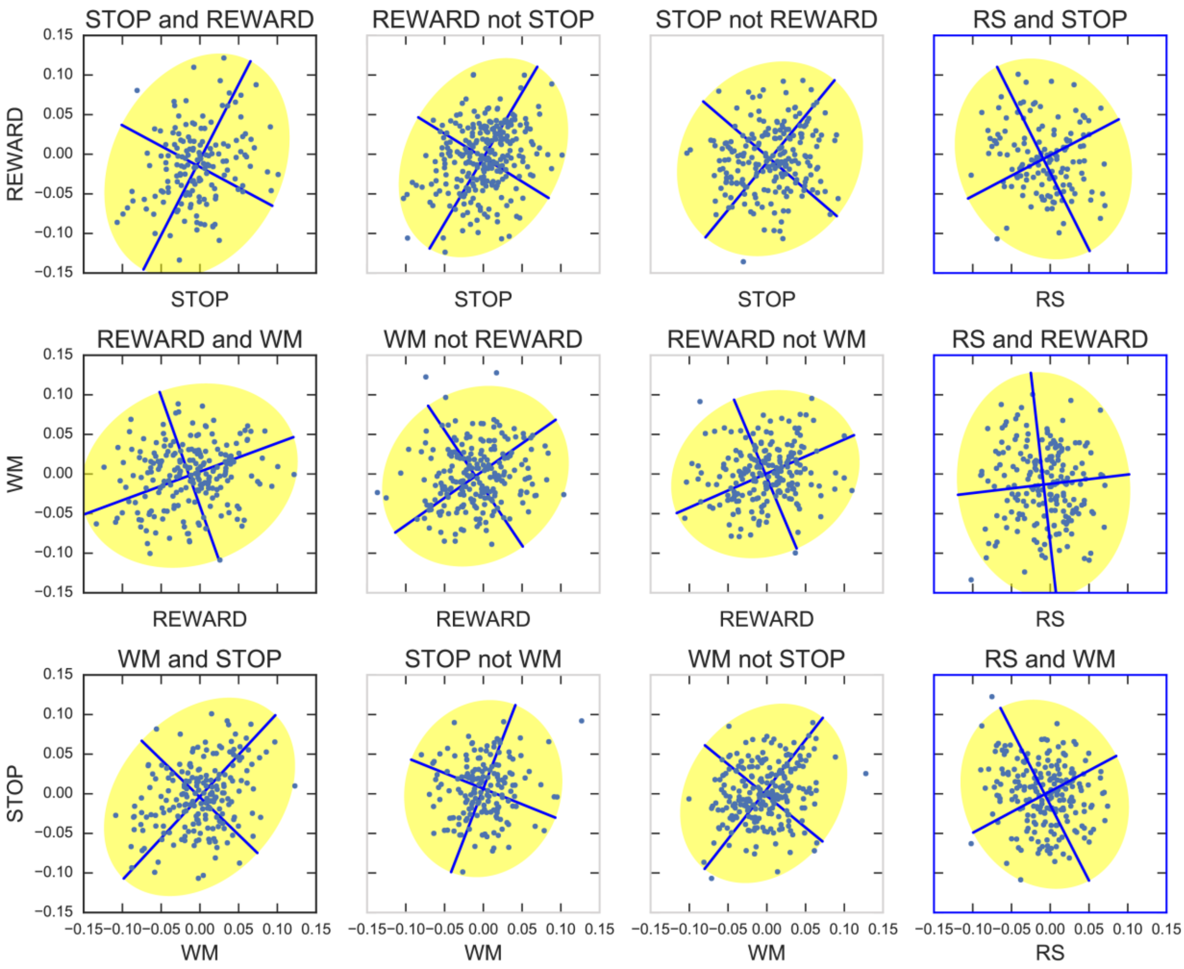

Supplementary Figure 6: Relationship of age effects between tasks for specific or common edges. A linear regression is computed against age, age-head motion interaction, and head motion for each edge in the task potency of each task. The beta parameters corresponding to the slope of the linear regression for the age effect are extracted for each edge and related between two tasks. Edges displayed in the left column are edges selected in both tasks included in the plot, the two central columns display correspondence for edges selected in only one of the two tasks of the plot. The right column displays correspondence for edges selected in the baseline fingerprint (i.e. the resting state *Z* partial correlation) versus one of the tasks. An ellipsoid is fit over the points in the scatter plot.

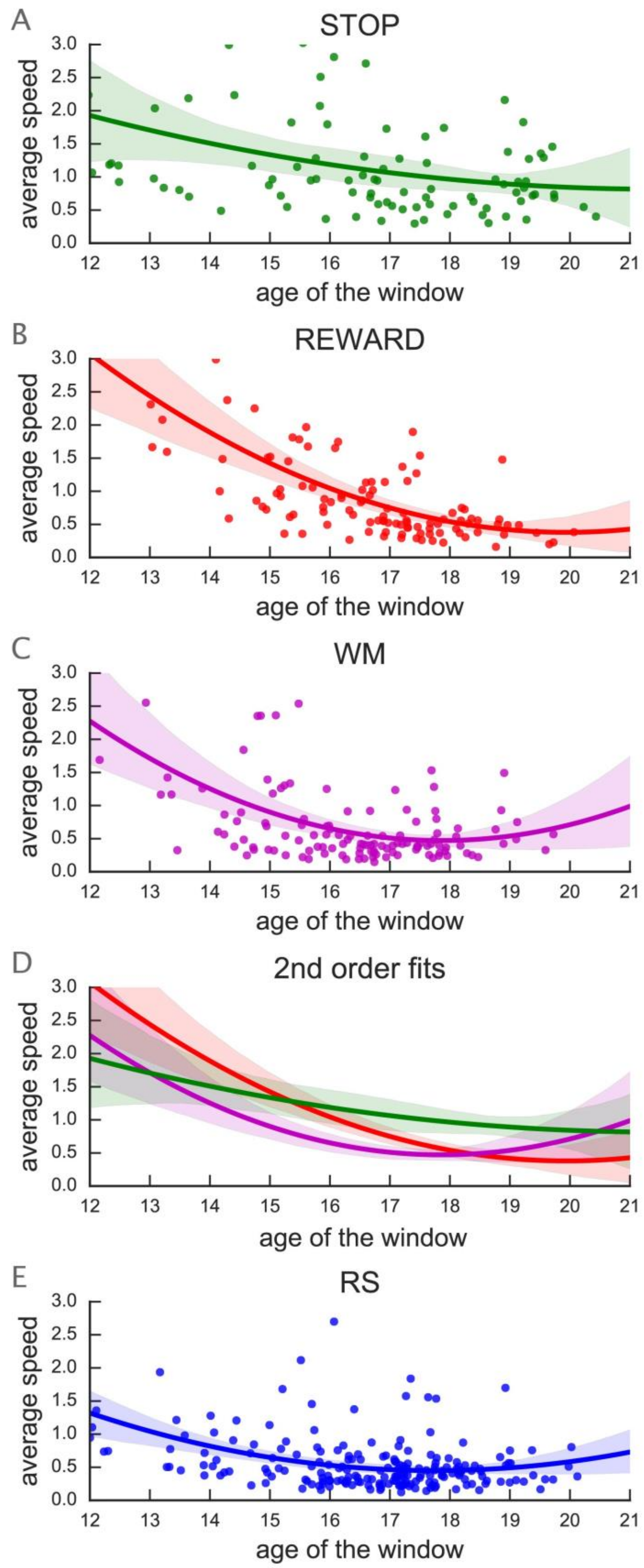

Supplementary Figure 7: Average speed of change with age of task potency for STOP (A), REWARD (B), and WM (C). Each plot illustrates the absolute beta-parameters relative to the age effect for each window in the sliding-average calculation using a linear model with age, age by head motion interaction, and head motion included in the model. For each task, we fit a 2nd order polynomial to model the rate of change across development. Graph D overlays each task's 2nd order fit to allow easy comparison between tasks. Finally, E illustrates the rate of change for RS.

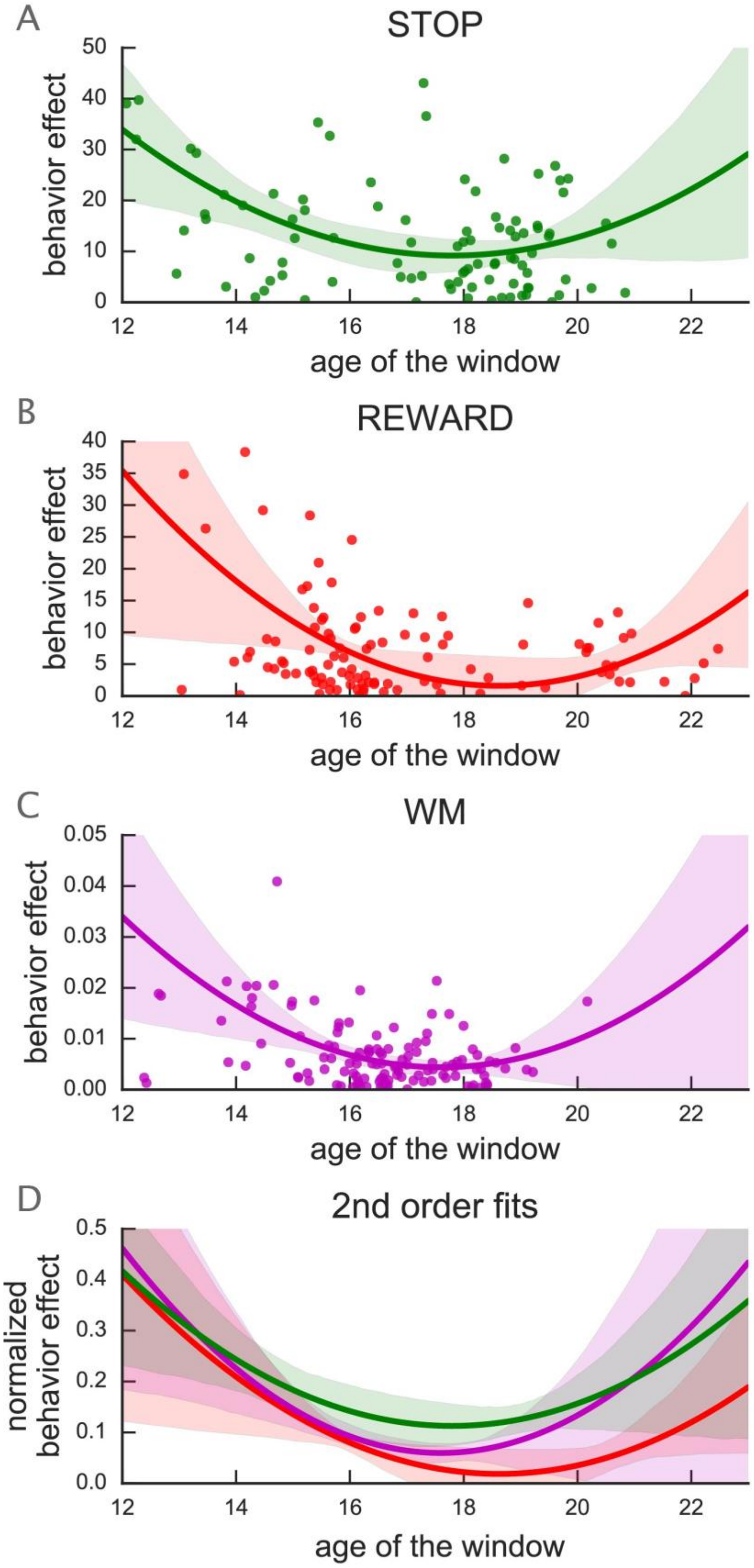

Supplementary Figure 8: Speed of change with age of task performance: stop signal reaction time (A), reward – non reward reaction time difference (B), and working memory performance (C). Each plot illustrates the absolute beta-parameters relative to the age effect for each window in the sliding-average calculation using a linear model. For each task, we fit a 2nd order polynomial to model the rate of change across development. Graph D overlays each task's 2nd order fit after normalization using the maximal beta of each task to allow comparison of dynamics between tasks.

3. References:

van Ewijk, H., Weeda, W.D., Heslenfeld, D.J., Luman, M., Hartman, C.A., Hoekstra, P.J., Faraone, S.V., Franke, B., Buitelaar, J.K., and Oosterlaan, J. (2015). Neural correlates of visuospatial working memory in attention-deficit/hyperactivity disorder and healthy controls. Psychiatry Res. Neuroimaging *233*, 233–242.

Hoogman, M., Aarts, E., Zwiers, M., Slaats-Willemse, D., Naber, M., Onnink, M., Cools, R., Kan, C., Buitelaar, J., and Franke, B. (2011). Nitric oxide synthase genotype modulation of impulsivity and ventral striatal activity in adult ADHD patients and healthy comparison subjects. Am. J. Psychiatry *168*, 1099–1106.

Jenkinson, M., Bannister, P., Brady, M., and Smith, S. (2002). Improved optimization for the robust and accurate linear registration and motion correction of brain images. NeuroImage *17*, 825–841.

Klingberg, T., Forssberg, H., and Westerberg, H. (2002). Training of Working Memory in Children With ADHD. J. Clin. Exp. Neuropsychol. *24*, 781–791.

Knutson, B., Fong, G.W., Adams, C.M., Varner, J.L., and Hommer, D. (2001). Dissociation of reward anticipation and outcome with event-related fMRI. Neuroreport *12*, 3683–3687.

Logan, G.D., Cowan, W.B., and Davis, K.A. (1984). On the ability to inhibit simple and choice reaction time responses: a model and a method. J. Exp. Psychol. Hum. Percept. Perform. *10*, 276–291.

McNab, F., Leroux, G., Strand, F., Thorell, L., Bergman, S., and Klingberg, T. (2008). Common and unique components of inhibition and working memory: An fMRI, within-subjects investigation. Neuropsychologia *46*, 2668–2682.

Rhein, D. von, Mennes, M., Ewijk, H. van, Groenman, A.P., Zwiers, M.P., Oosterlaan, J., Heslenfeld, D., Franke, B., Hoekstra, P.J., Faraone, S.V., et al. (2015). The NeuroIMAGE study: a prospective phenotypic, cognitive, genetic and MRI study in children with attention-deficit/hyperactivity disorder. Design and descriptives. Eur. Child Adolesc. Psychiatry 1–17.

von Rhein, D., Cools, R., Zwiers, M.P., van der Schaaf, M., Franke, B., Luman, M., Oosterlaan, J., Heslenfeld, D.J., Hoekstra, P.J., Hartman, C.A., et al. (2015). Increased Neural Responses to Reward in Adolescents and Young Adults With Attention-Deficit/Hyperactivity Disorder and Their Unaffected Siblings. J. Am. Acad. Child Adolesc. Psychiatry *54*, 394–402.

van Rooij, D., Hartman, C.A., Mennes, M., Oosterlaan, J., Franke, B., Rommelse, N., Heslenfeld, D., Faraone, S.V., Buitelaar, J.K., and Hoekstra, P.J. (2015). Altered neural connectivity during response inhibition in adolescents with attention-deficit/hyperactivity disorder and their unaffected siblings. NeuroImage Clin. *7*, 325–335.
